# Integrative NMR for biomolecular research

**DOI:** 10.1007/s10858-016-0029-x

**Published:** 2016-03-29

**Authors:** Woonghee Lee, Gabriel Cornilescu, Hesam Dashti, Hamid R. Eghbalnia, Marco Tonelli, William M. Westler, Samuel E. Butcher, Katherine A. Henzler-Wildman, John L. Markley

**Affiliations:** National Magnetic Resonance Facility at Madison and Biochemistry Department, University of Wisconsin-Madison, Madison, WI 53706 USA

**Keywords:** Automated spectral analysis, Automated structure determination and validation, Chemical shift assignment and validation, Peak identification, Restraint visualization and validation, Visualization of spectra, assignments, and structures

## Abstract

NMR spectroscopy is a powerful technique for determining structural and functional features of biomolecules in physiological solution as well as for observing their intermolecular interactions in real-time. However, complex steps associated with its practice have made the approach daunting for non-specialists. We introduce an NMR platform that makes biomolecular NMR spectroscopy much more accessible by integrating tools, databases, web services, and video tutorials that can be launched by simple installation of NMRFAM software packages or using a cross-platform virtual machine that can be run on any standard laptop or desktop computer. The software package can be downloaded freely from the NMRFAM software download page (http://pine.nmrfam.wisc.edu/download_packages.html), and detailed instructions are available from the Integrative NMR Video Tutorial page (http://pine.nmrfam.wisc.edu/integrative.html).

## Introduction

NMR spectroscopy is a powerful technique used in many areas of biomolecular research, including structural biology, enzymology, signal transduction, physiology, and drug discovery. NMR enables the collection of atomic-level data under conditions similar to those in cellular systems. Observable NMR parameters such as chemical shifts, peak intensities, scalar and dipolar couplings, line widths, and cross-relaxation provide critical information about target molecules and their interactions. An advantage of NMR, as one of the primary methods for structure determination, is its ability to detect local changes in conformation and dynamics that play functional biological roles.

Despite the growing number of facilities with NMR spectrometers operating at high magnetic fields, the approach has remained largely inaccessible to the larger biological community. In our experience, one reason is the steep learning curve required to become adept at acquiring, processing, and analyzing NMR data. For example, one needs to learn to tailor the experimental approaches and data analysis methods to the aims of the research. In addition, software packages commonly used require different computer operating systems and utilize different standards of atom nomenclature. The fragmentation of protocols presents a high barrier to entry into the field. The Collaborative Computing Project for NMR (CCPN, http://www.ccpn.ac.uk) took steps toward alleviating these problems through its development of *CCPNmr Analysis* (Vranken et al. 2005). In addition, the WeNMR project offers a number of relevant web-based resources for the process (Wassenaar et al. 2012). Nevertheless, these and other software resources fall short of covering the range of biomolecular experiments in current practice within an integrated package.

**Fig. 1:**
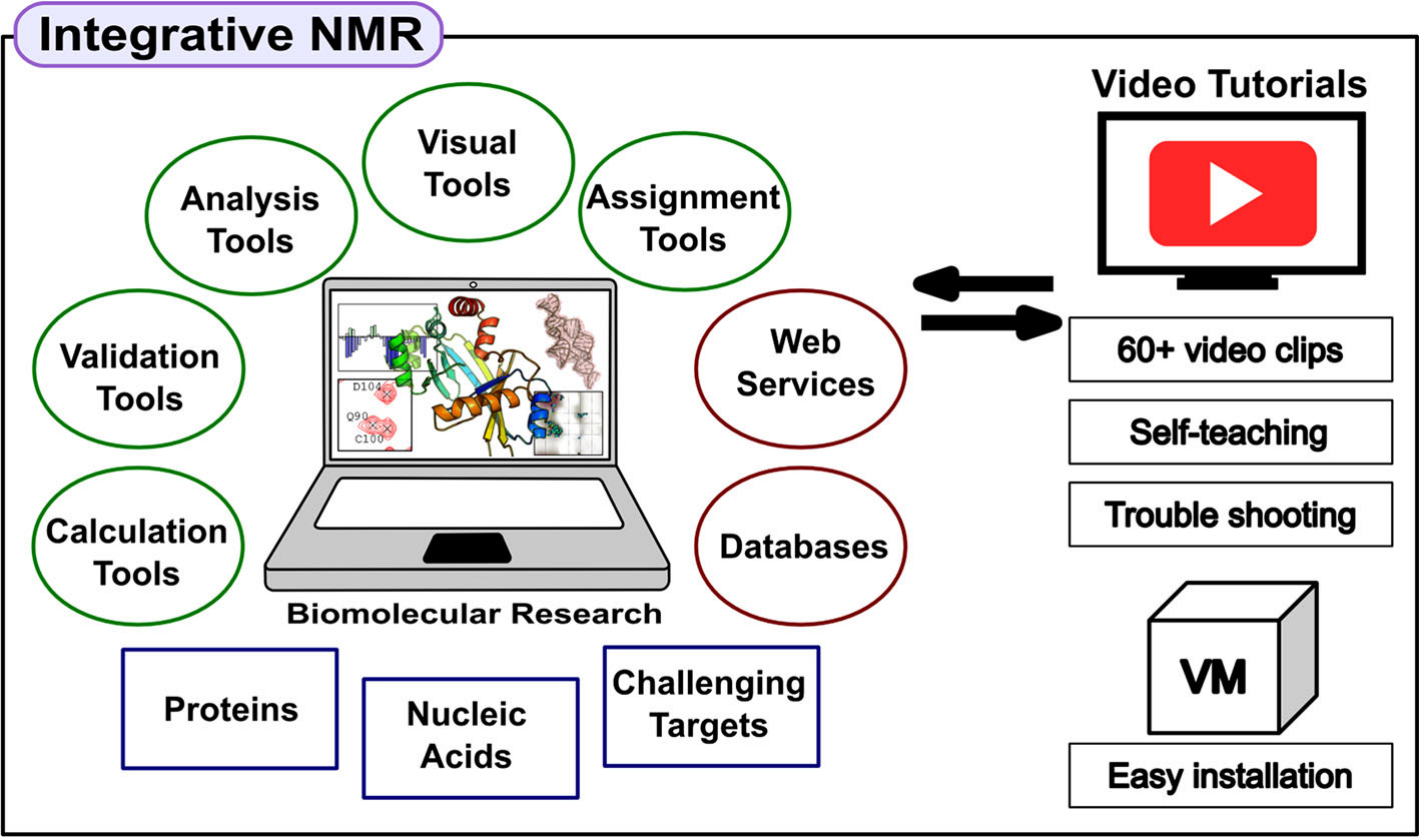
The *Integrative NMR* method for conducting biomolecular research. This integrated set of software packages, which can be installed on a laptop or desktop computer—optionally as part of a virtual machine, cover a wide range of data analysis and visualization steps in the workflows of biomolecular NMR research. The software tools interoperate seamlessly with external servers and databases. Video tutorials cover all operations

Our approach has been to develop software tools around the popular *Sparky* software package developed at the University of California, San Francisco (Goddard and Kneller 2008). We refined this platform through a series of nine annual workshops for neophytes held at the National Magnetic Resonance Facility at Madison (NMRFAM). Our objective was to establish a seamless, interactive environment for use by first-time users as well as practiced NMR spectroscopists. Within this platform, tasks are conducted by a series of freely available software packages, including those developed at NMRFAM. This approach has been refined through feedback from workshop students and worldwide users of these tools. The result of this effort is a software platform called *Integrative NMR* (Fig. 1), which makes biomolecular NMR spectroscopy much more accessible by integrating software tools so that they interact efficiently in ways that support both manual and automated approaches, result validation, and data visualization. Also included are links to web services, databases, and video tutorials. Although the component software packages are available for separate installation, we provide, as an option, all of them pre-installed in a virtual machine that can be run on any standard laptop or desktop computer. The virtual machine avoids the necessity of installing the separate required software programs within different operating systems.

Tasks are conducted by enhanced versions of two main software packages, *NMRFAM*-*SPARKY* (Lee et al. 2015) and *PONDEROSA* (Lee et al. 2011, 2014) in which old and new tools are integrated in efficient ways that emphasize visualization. For example, the *Dummy Graph* tool in *NMRFAM*-*SPARKY* depicts regions of the covalent structure of proteins or DNA/RNA molecules along with the status of current chemical shift assignments of their NMR-active atoms. RNA assignments are facilitated by ellipses drawn over spectra to delineate statistical chemical shift assignment regions for atoms in particular bases (Aeschbacher et al. 2013). Experimental data from spectral series, such as pH titrations, molecular interaction studies, or NMR relaxation, can be visualized seamlessly with the *NDPPlot* (NMR Data Perturbation Plot) tool in *NMRFAM*-*SPARKY.* New visual analysis tools in Ponderosa Analyzer simplify many time-consuming tasks to a few screen clicks. An enhanced mode of the *PyMOL* software package (The PyMOL Molecular Graphics System, Version 1.7.4 Schrödinger, LLC.), which supports shortcut commands, enables the visualization of data from the analysis and validation packages of *Ponderosa Analyzer*. With the virtual machine, a user can launch the calculation of the structure of a protein from NMR data by a few clicks. Without the need to install any individual software packages, the process can make use of *APES* for peak picking (Shin et al. 2008), *PINE* for automated assignment (Bahrami et al. 2009), *PONDEROSA*-*C/S* for automated structure determination, *TALOS*-*N* for shift based torsion angle restraints (Shen and Bax 2013), the *PACSY* database (Lee et al. 2012), and the *CS-Rosetta* (Lange et al. 2012) compute server at the Biological Magnetic Resonance data Bank (BMRB). Furthermore, all automated approaches are accompanied by efficient visual verification tools: automated peak picking can be verified by a tool in *NMRFAM*-*SPARKY*; errors in automated assignments by *PINE* can be detected and corrected with *PINE*-*SPARKY* (Lee et al. 2009) or *ARECA* (Dashti et al. 2016); and errors in automated structure calculations can be detected and corrected by the visual tools that are part of *Ponderosa Analyzer*.

A goal of Integrative NMR is to incorporate multiple approaches to the solution of problems as proposed recently (Dashti et al. 2015). For example, peak assignments can be carried out manually or automatically and NOE peaks can be picked manually or automatically. As its default, *PONDEROSA* utilizes the *Xplor*-*NIH* engine for structure determination, but users with a license for *CYANA* can use the *CYANA* engine as an option along with its automated NOE assignment module. The default tools are designed to work with well-folded proteins of small or moderate size. *NMRFAM*-*SPARKY* contains additional tools that are useful for larger proteins or intrinsically disordered proteins. The developers of *SCAssign* (Zhang and Yang 2006) for the assignment of larger proteins and *ncIDP*-*assign* (Tamiola and Mulder 2011) for intrinsically disordered proteins have permitted their inclusion in *NMRFAM*-*SPARKY*.

All software, including the virtual machine, is freely available from the NMRFAM website (http://pine.nmrfam.wisc.edu/download_packages.html), and video tutorials available from the website cover every step.

## Materials and methods

The *Integrative NMR* platform makes use of several software packages developed at the National Magnetic Resonance Facility at Madison (NMRFAM) and elsewhere. The software packages can be installed separately or can be obtained from NMRFAM installed on a virtual machine that can be used on a variety of computer platforms. This latter approach, which does not entail significantly longer software run times, is particularly useful for non-specialists. The platform provides user-friendly interfaces to freely-available servers in the biomolecular NMR field.

### *NMRFAM*-*SPARKY* and its tools

The originators of *Sparky* transferred the development of this popular software package to NMRFAM. We modernized and enlarged many parts of the core engine written in *C*++ with extensions in *Python*, added new tools that integrate freely-available tools in the biomolecular NMR field, and released the new version as *NMRFAM*-*SPARKY* (Lee et al. 2015). For the benefit of legacy users, we kept changes in user interfaces to a minimum. Continued development described here has focused on the addition of new features and their graphical interfaces and on seamless integration with relevant web services. The tools are menu driven, but *Integrative NMR* supports many shortcut two-letter commands that more conveniently activate individual tools within *NMRFAM*-*SPARKY* (Table 1).

**Table 1:** Two-letter-codes (case sensitive) used within *NMRFAM*-*SPARKY* to activate tools that carryout various operations in Integrative NMR

Peak identification	
*ae*	*APES* automated peak picking. Peak positions are identified from local maxima, and peak positions in multiple spectra are compared to flag peaks that are not part of spin systems as noise. Potential noise peaks can be identified and deleted automatically
*kr*	Restricted peak picking. Peaks are identified on the basis of local maxima within search windows specified by peaks in another spectrum
*LT*	Alternate peak list window. Peaks identified from local maxima are sorted by data height; this helps to identify noise peaks which are often have low intensity
*sp*	Strip plot. Once peaks have been identified and noise peaks have been eliminated, the strip plot tool can be used to efficiently delete any remaining false-positive peaks and add missing peaks
Automated protein chemical shift assignment	
*ep*	*PINE* automated assignment. This bring up a window that can be used to specify peak lists from different NMR experiments and launch a submission to the *PINE Server* to carry out automated protein peak assignments
*ip*	Convert *PINE* outputs to Sparky. This tool converts probabilistic backbone and sidechain assignment files generated by the *PINE Server* to a Sparky resonance file that can be read in by two-letter-code *rl* with the probability set manually
*rl*	Resonance list. This window shows currently assigned resonances with averaged chemical shifts and their deviations. In *Integrative NMR*, this tool is used to read-in/write-out chemical shifts
*p2*	*PINE2SPARKY* converter. *PINE2SPARKY* generates probable candidates for all peak in the spectra prior to using *PINE*-*SPARKY* to verify the *PINE* output against spectra
*ab*	*Assign the Best by PINE*. After using *PINE2SPARKY* to import the probabilistic assignments from *PINE* to *NMRFAM*-*SPARKY*, this tool can be used to set a threshold and to accept all assignments with probabilities that exceed this threshold
*pp*	*PINE Graph Assigner*. This tool enables graphical examination of all probable assignment candidates on a per-residue and atom-by-atom basis
*pr*	*PINE Assigner*. This tool enables the examination of all assignment candidates on a peak-by-peak basis
Enhanced manual protein chemical shift assignment	
*ta*	*Transfer and Simulated Assignments.* This versatile tool annotates peaks on a selected spectrum on the basis of assignments from other spectra or predictions. If the assignment is simulated from prediction, the assignment tag contains “_s” to avoid confusion
*ut*	Untag “_s”. This command detaches “_s” from a selected tag for a peak whose assignment has been confirmed
*cu*	Center and Untag “_s”. This command causes a peak identifier to move to the nearest local maximum and detaches the “_s” tag
*mt*	Merge two assignments to a pseudoatom. If two assignments are overlapped after centering and untagging by use of the *cu* command, the user can merge them as one pseudoatom by typing the *mt* command
Chemical shift validation	
*lv*	Run *LACS*. This command submits a protein chemical shift file for analysis by *LACS* (Linear Analysis of Chemical Shifts); the LACS output detects chemical shift outliers and detects chemical shift referencing errors and suggests chemical shift corrections
*ea*	Generate files and export to *ARECA*. This command opens a window that enables the generation of ARECA input files (peak assignments and NOE peak lists) and opens the *ARECA* web page to import the files and launch *ARECA* to validate the assignments
*ar*	*ARECA* list. This tool enables the user to color peaks and assignments in 3D-NOE spectra according to the assignment probabilities generated by *ARECA* as a means for their validation
Molecular structure visualization	
*dg*	*Dummy Graph*. This command launches a molecular structure visualization tool that shows the atoms and their assignment status
Tools for intrinsically disordered proteins (IDPs) and large proteins	
*RS*	*ncIDP Repositioner*. Repositions an assigned stretch of protein sequence according to *ncIDP* chemical shift statistics
*SG*	*ncIDP Spin Graph*. Spin graph modified for intrinsically disordered proteins (IDPs)
*sn*	*SCAssign*. Sidechain assignments from 4D-NOESY and CCH-TOCSY data
Nucleic acid assignment	
*ER*	Export to *RNA*-*PAIRS*. This tool generates *RNA*-*PAIRS* inputs and opens the web page of the *RNA*-*PAIRS* server
*SE*	RNA statistical ellipses. Draws ellipses on 2D spectra that delineate the ranges of chemical shifts expected for particular RNA bases in the *CHESS2FLYA* program
*DG*	*Dummy Graph* for nucleic acids. This tool displays atoms from the covalent structures of DNA/RNA residues and indicates the current status of chemical shift assignments
Spectral series	
*ol*	View overlays. Overlays NMR spectra for comparison
*ct*	Color contour levels. This tool enables the user to differentially color the contour plots from overlaid 2D NMR spectra
*np*	Perturbation plot. This tool enables the user to construct plots that compare specified NMR observables from two spectra collected under different conditions
*ni*	Titration plot. This tool traces changes in the chemical shift of a particular resonance in multiple spectra as the function of a variable such as pH or added ligand
*rh*	Peak height analysis. This command enables the plotting of peak heights as a function of assigned residue number or by corresponding resonances in different spectra. The changes in peak height can be saved in tabular form for further analysis. A decaying exponential function is also fit to the data For analysis of *T*_1_/*T*_2_ relaxation data, the peak heights can be fitted to a decaying exponential function. The extracted relaxation constants can then be plotted as a function of residue number
*eo*	Easy overlay dialog. Enables users to easily overlay NMR spectra by a few clicks
*ec*	Easy contour dialog. Enables users to easily adjust contour levels of NMR spectra by a few clicks.
*ci*	Inverse background color. This command changes background color from black to white or from white to black
Secondary structure prediction	
*n6*	*PECAN*. This command uses assigned chemical shifts as input to *PECAN*, which carries out probabilistic chemical shift based secondary structure prediction
*tl*	*TALOS*-*N*. This command uses assigned chemical shifts as input to *TALOS*-*N* ^,^ which carries out artificial neural network chemical shift based secondary structure prediction
*PP*	*PSIPRED*. This command uses amino acid sequence as input to *PSIPRED*, which carries out Psi-blast sequence based secondary structure prediction
Three-dimensional structure prediction	
*nm*	*POND*-*PRED* (*Ponderosa Prediction Server*). This command invokes this server that predicts 3D structure on the basis of amino acid sequence alone. The server uses hydrogen bond constraints from secondary structure predicted by *PSIPRED*, and distance and angle constraints from the *PACSY* database to generate structures by simulated annealing from Ponderosa Server
*ce*	*CS*-*Rosetta*. This command brings up the BMRB-hosted 3D structure prediction server based on Monte Carlo assembly with chemical shift filtered protein fragments
Three-dimensional structure determination	
*c3*	*PONDEROSA*-*C/S* structure calculation. This command carries out automated NOESY peak picking to generate the input for the *Ponderosa Server* at NMRFAM, which then calculates the 3D structure of the protein
*cp*	*Ponderosa Client*. This command launches the *Ponderosa Client* program that enables the specification of additional input for 3D structure calculation, including RDC, SAXS, WAXS, and the use of alternative calculation methods
*up*	Ponderosa Connector. This command establishes a connection between *PONDEROSA*-*C/S* and *NMRFAM*-*SPARKY* that enables interactive assessment of NOESY peak quality and validation of distance constraints. PONDEROSA-C/S specifies regions of interest to *NMRFAM*-*SPARKY*, which displays spectra so that users can decide whether peaks are real and assignments are valid
*gd*	Generate distance constraints. This tool uses the *r* ^*−*3^–*r* ^*−*6^ approximation to automatically generate distance constraints in *PONDEROSA* compatible format (*DYANA*) from assigned NOE cross peaks
*xf*	Manual restraint format. This tool uses a manual binning approach based, as specified, either on peak height or volume to generate distance constraints in *PONDEROSA* or *XPLOR* compatible format from assigned NOE cross peaks

### NDPPlot (NMR data perturbation plot)

A feature lacking in the original *Sparky* software was data visualization from experiments producing spectral series, such as NMR relaxation or titration studies. In order to add a chart plotting tool that works seamless with *NMRFAM*-*SPARKY*, we chose *Free Pascal* and *Lazarus IDE* (http://www.lazarus-ide.org) for its development because of their convenience in producing statically compiled executable binaries in Windows, Mac, and Linux and because of our prior experience with this IDE (integrated development environment) in developing the *Pine2Sparky* converter (Lee et al. 2009). The new graphical plotting program is called *NDPPlot* (NMR data perturbation plot); although it was developed initially for chemical shift tracing, it has proved to be versatile for use in other applications.

### Structure calculation

The structure calculation server program, *Ponderosa Server*, and the NOESY peak picking and data transfer program on the client side, *Ponderosa Client*, are written in *C*++ with *QT* libraries (http://www.qt.io). We developed an interface between the *PACSY* database and *Ponderosa Server* to support the *AUDANA* algorithm (Automated Database-Assisted NOESY Assignment) for automated structure calculation (Lee et al., submitted) and the *PACSY*-*ALIGN* algorithm for finding similarities within the protein database (http://pacsy.nmrfam.wisc.edu/pacsyalign). We wrote *Xplor*-*NIH* scripts (Schwieters et al. 2003) for structure calculation in *Python*. We wrote *NMRFAM*-*SPARKYPython* extension codes for *PONDEROSA*-*C/S* interface that make processes flawless. Furthermore, we built web server for public services with *HTML*, *Apache*, *CGI*, *Perl*, *Python*, and *MySQL* on our Linux cluster system. We prepared 256 CPU cores as structure calculation resources at NMRFAM. We added advanced structural analysis tools (written in *Free Pascal* with *Lazarus IDE*) to *Ponderosa Analyzer*, the program that validates results from structural calculations and assists with iterative calculations. In addition, we created interfaces linking *Ponderosa Analyzer*, *NMRFAM*-*SPARKY*, and *PyMOL*.

### Sample data

NMR data for ubiquitin, SIV, and NANOG were acquired at NMRFAM; data for UbcH5B/CNOT4 was from Dr. A.M.J.J. Bonvin’s web page (http://www.nmr.chem.uu.nl/~abonvin/); and data for OR135 was from the CASD-NMR web page (https://www.wenmr.eu/wenmr/casd-nmr). We used data from ubiquitin (unpublished) and SIV frameshift site RNA (Marcheschi et al. 2007) to develop tools, respectively, for general spectral analysis and assignment of proteins and RNA molecules. We used data from NANOG (unpublished) to develop tools for peak height analysis, UbcH5B/CNOT4 (Dominguez et al. 2004) to develop tools for perturbation/titration analysis, and from OR135 (Rosato et al. 2015) to develop of structure calculation tools (Koga et al. 2012).

### Video tutorials

Videos were recorded in *OGV* format by *RecordMyDesktop* software (http://recordmydesktop.sourceforge.net), converted to *MKV*-formatted files, and uploaded onto *YouTube* (http://www.youtube.com) with added annotations to explain features. Videos can be accessed from (http://pine.nmrfam.wisc.edu/integrative.html); users are encouraged to subscribe to the YouTube channel to receive notifications of uploads of new video tutorials.

### Installation of separate modules

We provide simple installers for the software components of *Integrative NMR* on all supported platforms (*Python* for Linux and Mac, and *Windows Batch* for Windows).

### Virtual machine

In addition, we make all the software components of *Integrative NMR* available on a virtual machine. An ISO-formatted 64-bit disk image of Ubuntu MATE 15.04 was downloaded from the Ubuntu MATE web page (http://ubuntu-mate.org) and installed in an *ORACLE VM VirtualBox* (http://www.virtualbox.org). The software components of *Integrative NMR* were installed and optimized on this virtual machine. Then, the virtual disk image was exported to Open Virtualization Archive (OVA) format. In addition, we used the *7*-*zip* file compression program (http://www.7-zip.org) to prepare a separated compressed version of the virtual machine for 32-bit operating systems that cannot download files larger than 2 GB from a web browser.

## Results and discussion

### *NMRFAM*-*SPARKY* and its tools

#### Peak identification

**Fig. 2:**
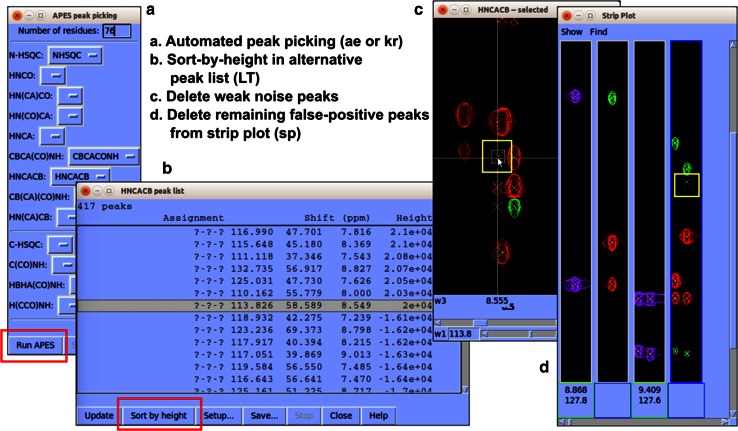
Recommended semi-automated peak identification method in *NMRFAM*-*SPARKY*. **a** Use APES (two-letter-code *ae*) or restricted peak picking (two-letter-code *kr*) for automated peak identification. **b** Use alternative peak list window (two-letter-code *LT*) to sort peaks by intensities. **c** Delete weak noise peaks from spectrum view. **d** Use strip plot (two-letter-code *sp*) to delete any remaining false-positive peaks and add missing peaks

The basic approach to peak identification in 2D biomolecular NMR spectra is to search for local maxima above a chosen contour level. If a graphical tool is used to select the peaks, this algorithm is generally successful; however, when peak picking is automated, too many noise peaks can be included. With spectra of dimension greater than two, visual searching, becomes highly time consuming. Therefore, it is common to use a visual peak picking tool to identify peaks in 2D HSQC spectra first and to use automated peak picking restricted to the chosen frequencies to identify peaks in 3D spectra. As with 2D spectra, the automated approach can include noise and artifacts. To get around this problem *NMRFAM*-*SPARKY* employs two advanced automated restricted peak picking tools: *APES* (Shin et al. 2008) and *PONDEROSA*. With these tools, one can utilize an alternative peak list window (two-letter-code *LT*) and strip plot window (two-letter-code *sp*) to complete the peak picking step as illustrated in Fig. 2.

#### Automated protein chemical shift assignment

The *Integrative NMR* suite includes the *PINE* (Bahrami et al. 2009) assignment engine (two-letter-code *ep*), which supports probabilistic backbone and sidechain assignments based on available NMR data sets. The ranked assignments proposed by *PINE* are easily validated and extended through the use of *PINE*-*SPARKY* (two-letter-codes *ip, p2,ab*, *pp*, and *pr*), which enables the visualization of proposed assignments against experimental spectral data (Lee et al. 2009).

#### Enhanced manual protein chemical shift assignment

**Fig. 3:**
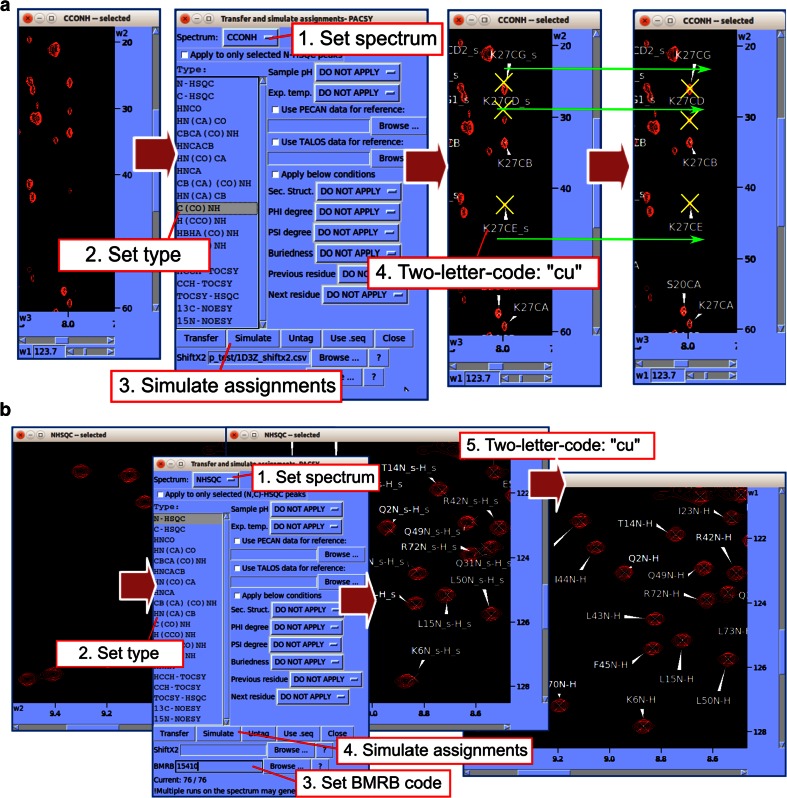
*Predict*-*and*-*confirm* method for fast semi-automated chemical shift assignment. **a** Efficient method for assigning a C(CO)NH spectrum. In the *Transfer and simulate assignment* window (two-letter-code: ta), set *Spectrum* and *Type*; then click the *Simulate* button to annotate predicted assignments on the experimental spectrum (yellow ‘X’s). Drag each yellow X onto the nearest peak in the spectrum, and type “cu” to confirm the assignment. The yellow ‘X’s are now centered on the position of the experimental peak. **b** Illustration of how *Predict*-*and*-*confirm* can be used to import assignments from a BMRB entry. The BMRB assignments are displayed over the spectrum, and the user can then adjust and confirm them

*Transfer and Simulated Assignments* (two-letter-code *ta*) is a versatile assignment tool recently developed under *NMRFAM*-*SPARKY* that uses the *PACSY* database (Lee et al. 2012) to enable a new assignment method, *predict*-*and*-*confirm*. This approach greatly accelerates assignments by eliminating the redundant procedures and potential user errors associated with the traditional *pick*-*and*-*assign* method. *Transfer and Simulated Assignments* was originally devised for fast side chain assignment from spectra such as C(CO)NH, H(CCO)NH, and HBHA(CO)NH (Fig. 3a); however, as shown in Fig. 3b, if a corresponding BMRB entry exists, the approach can be used for one-shot assignments based entirely on 2D HSQC spectra.

#### Chemical shift validation

Linear Analysis of Chemical Shifts (*LACS*) is supported by NMRFAM-SPARKY (two-letter-code *lv*); LACS detects and corrects errors in chemical shift referencing (Wang et al. 2005). *ARECA* (Assessment of the REliability of Chemical shift Assignments) is a tool for validating protein chemical shift assignments on the basis of NOE data (Dashti et al. 2016). The input can be prepared by either *NMRFAM*-*SPARKY* (two-letter-code *ea*) or *Ponderosa Client* from ^15^N- and/or ^13^C-filtered NOE experiments (two-letter-code *pc*). The *NMRFAM*-*SPARKY* extension for *ARECA* (two-letter-code *ar*) handles data analysis. Chemical shifts can be validated in advance of a structure determination to minimize subsequent refinement steps. Validated assignments are also important for other types of experiments, such as ligand binding or dynamics studies.

#### Molecular structure visualization

**Fig. 4:**
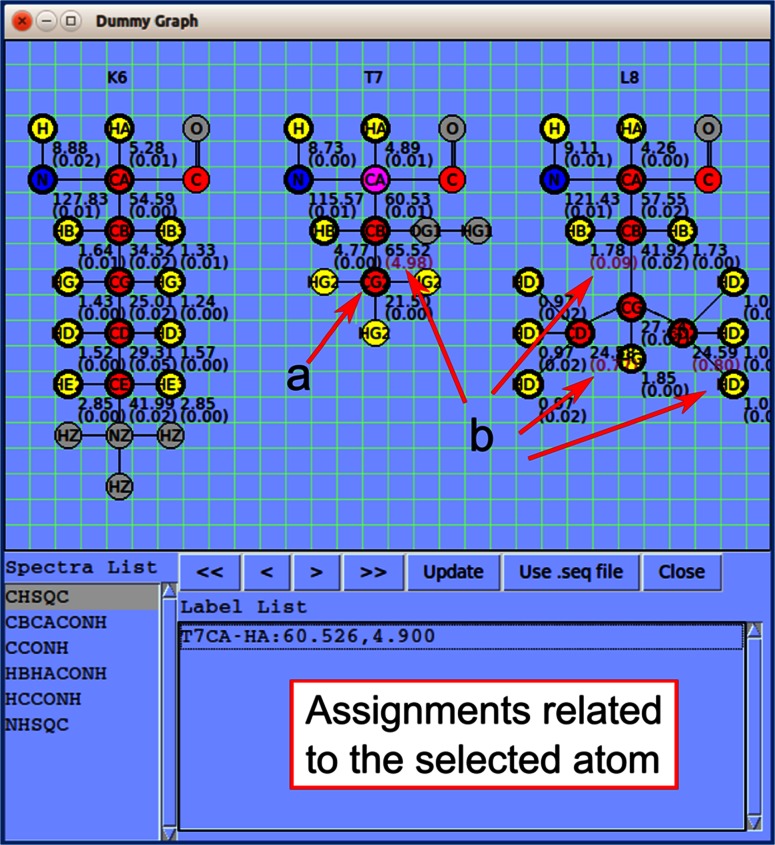
*Dummy Graph* is molecular structure visualization tool that shows the atoms in a three-residue stretch and indicates which atoms have been assigned. Also shown is the average chemical shift and standard deviation (in ppm) from the positions of all peaks in the different spectra supporting a given assignment. An atom can be selected by clicking; this turns its *background pink* as shown for T7CA. The *panel at the bottom* shows assignments (in the *Label List*) related to the selected atom from the spectrum selected in the *Spectra List*. **a** A *thick line* *around an atom* and *chemical shift* indicates that the atom is already assigned. Otherwise, a *thin line around an atom* indicates that it is not yet assigned. **b** Large chemical shift deviations are written in *purple* to alert the user to the possibility of an erroneous assignment

*Pine Graph Assigner*, the visual tool for molecular structure visualization in the original *PINE*-*SPARKY* (Lee et al. 2009), has been simplified and generalized for universal use as *Dummy Graph* (Fig. 4, two-letter-code *dg*). *Dummy Graph* shows atoms to be assigned along with average and standard deviation of assigned chemical shifts; it also shows the assignment labels for a selected atom and enables the user to visualize the place in a given spectrum where the assigned peak is located. Missing assignments (Fig. 4a) and erroneous assignments (Fig. 4b) can be recognized by direct visualization.

#### Tools for intrinsically disordered proteins (IDPs) and large proteins

*NMRFAM*-*SPAR*KY supports the assignment of challenging targets such as IDPs and large proteins. For IDP assignment, *NMRFAM*-*SPARKY* includes the set of tools developed by Mulder group including their IDP chemical shift statistics (Tamiola and Mulder 2011). The *ncIDP*-assign package, which consists of *ncIDP Repositioner* (two-letter-code *RS*) and *ncIDP Spin Graph* (two-letter-code *SG*), is pre-installed. For large proteins, the SCAssign package (two-letter-code *sn*) supports assignments based on 4D ^13^C-,^15^N-edited NOESY and 3D CCH-TOCSY spectra (Zhang and Yang 2006). See http://yangdw.science.nus.edu.sg/SCAssign for an online tutorial from the Yang group. These approaches become more powerful within *NMRFAM*-*SPARKY* because they can take advantage of the *predict*-*and*-*confirm* and *Dummy Graph* methods described above.

#### Tracking of manual assignments

*NMRFAM*-*SPARKY* supports the annotation module of *CONNJUR R* (Fenwick et al. 2015), which records information about peaks that have been reassigned manually. This functionality can be used to improve the reproducibility of NMR structure determinations. This feature is available on the virtual machine, Linux, and Mac versions of *Integrative NMR*.

#### Nucleic acid assignment

**Fig. 5:**
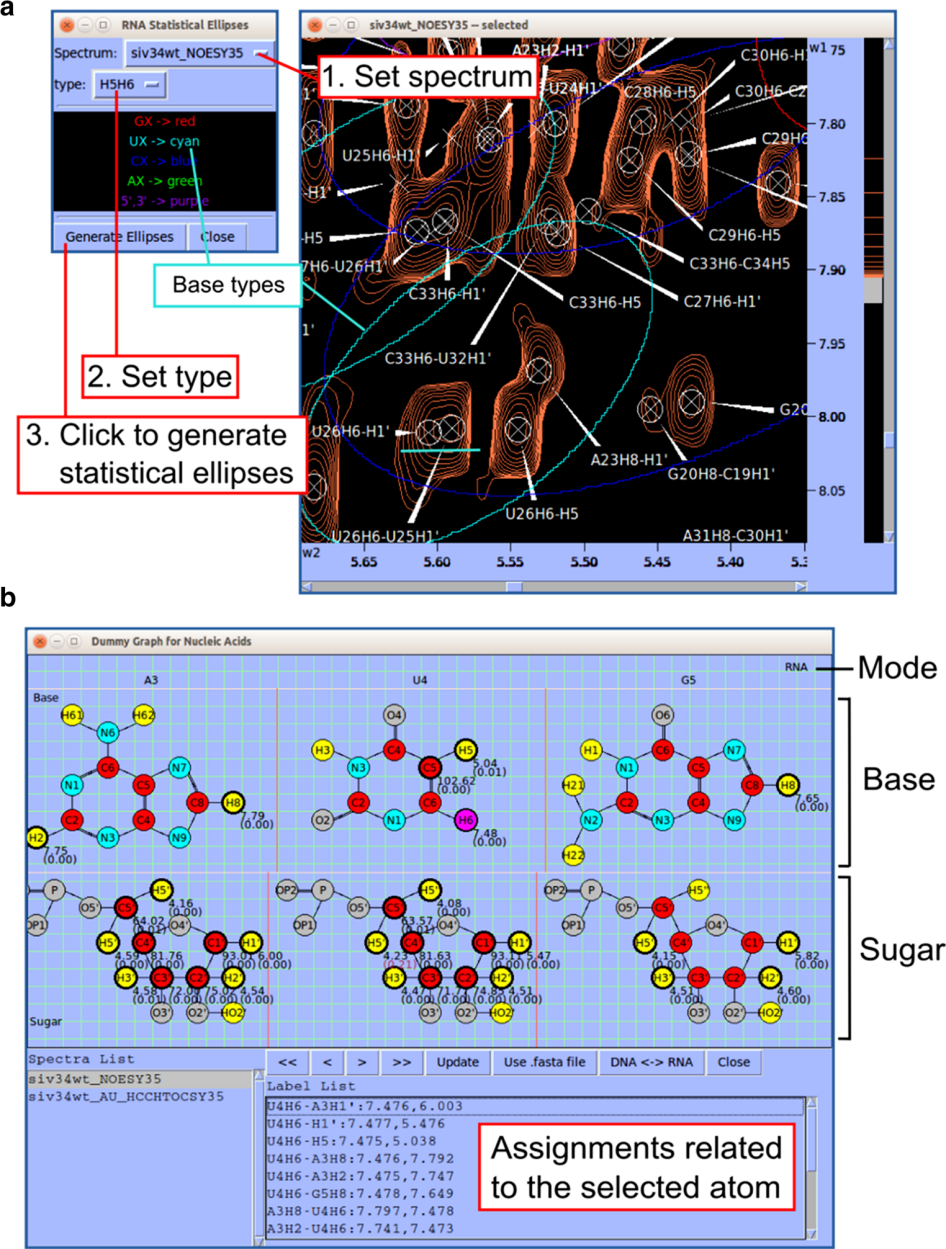
Tools developed for assisting chemical shift assignment of nucleic acids. **a** *NMRFAM*-*SPARKY* supports covariance ellipses for RNA ^1^H and ^13^C chemical shifts first introduced in the *CHESS2FLYA* program. *Statistical ellipses* is a tool in *NMRFAM*-*SPARKY* (two-letter-code *SE*) that overlays *color-coded ellipses* on the spectra to outline the range of chemical shifts expected for different RNA bases. The peaks *underlined* in *cyan* are near the center of the cyan ellipse, which corresponds to Uracil. **b** *Dummy Graph for Nucleic Acids* (two-letter-code *DG*) is a tool for visualizing atoms of DNA or RNA residues; base atoms of are shown at the *top* and those of the sugar and phosphate are shown at the *bottom.* The *panel below* shows assignments related to the selected atom shown in *pink* (U4H6) from the selected spectrum

*RNA*-*PAIRS* is an algorithm for automated RNA imino resonance assignment (Bahrami et al. 2012). *NMRFAM*-*SPARKY* contains a link (two-letter-code *ER*) that generates RNA-PAIRS inputs and redirects the user’s web browser to the *RNA*-*PAIRS* web server page at NMRFAM. RNA chemical shift statistics calculated by the Schubert group (Aeschbacher et al. 2013) suggested covariance statistics for ^1^H and ^13^C chemical shifts. The *RNA Statistical Ellipses* window in *NMRFAM*-*SPARKY* (Fig. 5a) displays the statistical ellipses overlaid on RNA spectra to assist chemical shift assignment. A nucleic acid version of *Dummy Graph* (Fig. 5b) displays the atomic structure of the DNA or RNA molecule being assigned.

#### Spectral series

**Fig. 6:**
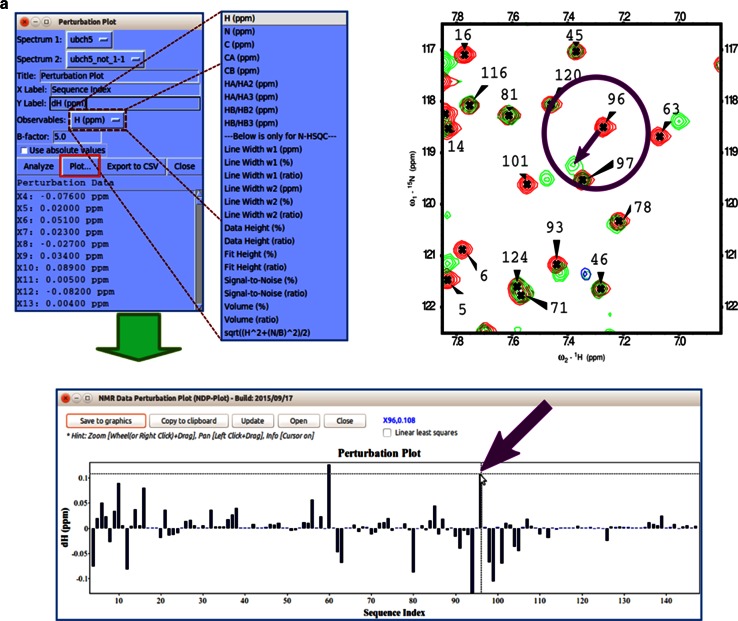

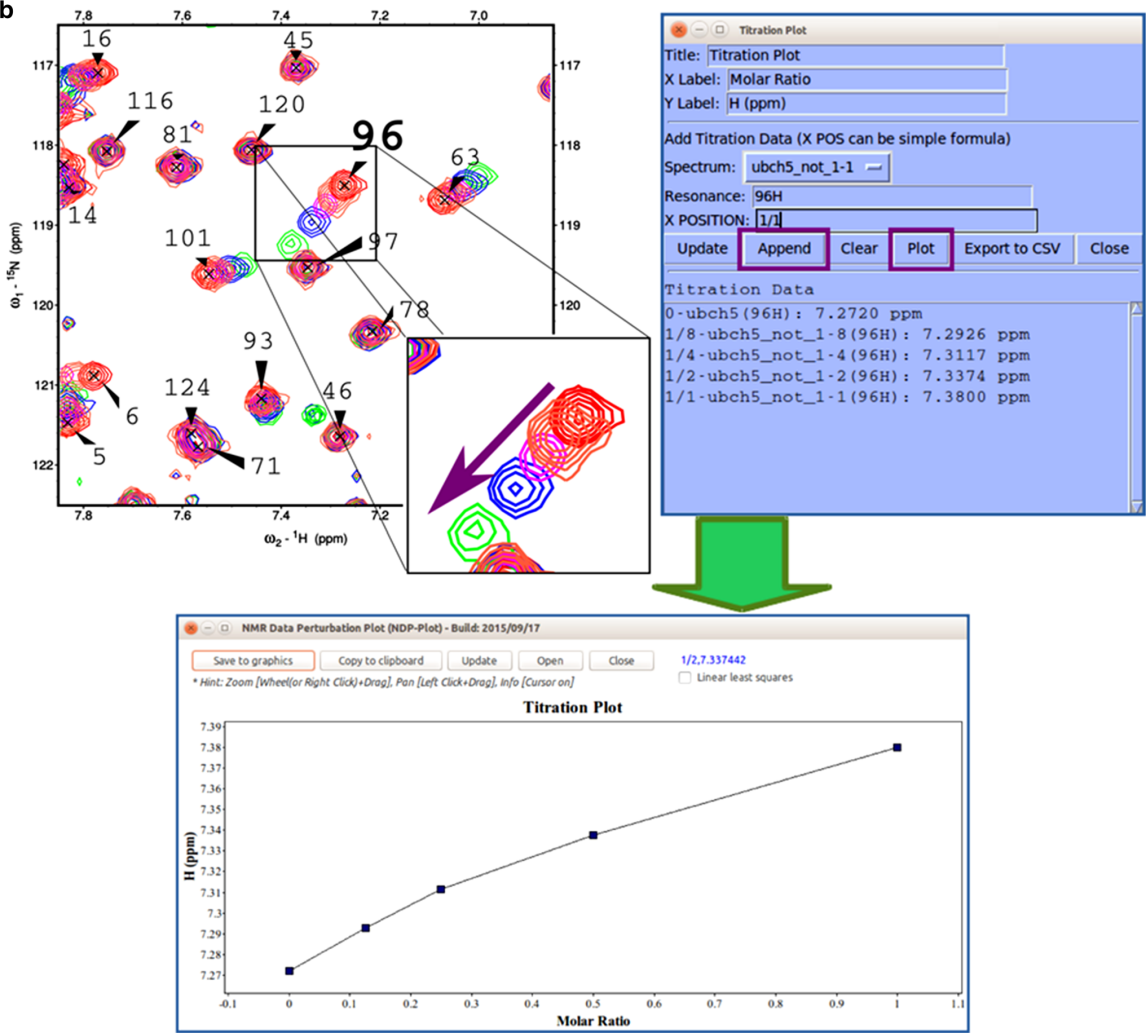

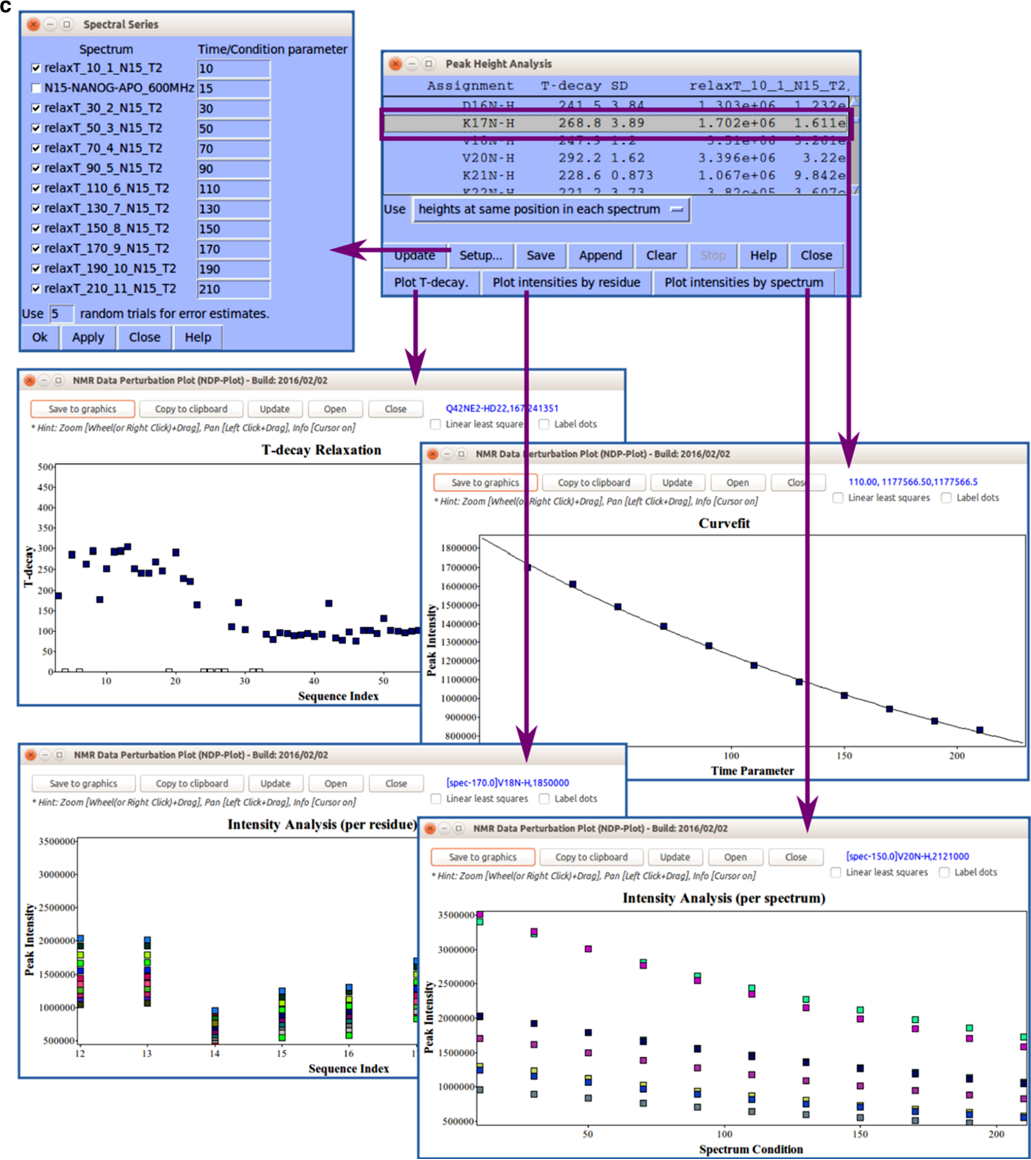
*NDPPlot* is a tool fully integrated into *NMRFAM*-*SPARKY* for visualizing spectral changes. **a** *Perturbation plot* (two-letter-code *np*). The *arrows point* to the change in the chemical shift of residue 96. The *contour plot* overlays (two-letter-code *ol* or *eo*) two selected ^1^H–^15^N HSQC spectra: one recorded with (*green contour*) and one without (*red contour*) added substrate. The displacement of the signal assigned to residue 96 is highlighted within the *circle*. *NDPPlot* generates the bar chart shown to plot the chemical shift differences between two spectra along the sequence; this is achieved by choosing the two spectra and the observable to be compared (in this case, the chemical shift of amide protons) in the *Perturbation plot* window and by clicking *Plot*. **b** *Titration Plot* (two-letter-code *ni*) visualizes chemical shift changes from a titration experiment. Results are plotted by the *NDPPlot* program. This example shows how the ^1^H NMR chemical shift of residue 96 from the ^1^H-^15^N HSQC spectra changes upon the addition of a substrate. The spectrum shows the overlap (two-letter-code *ol*) of spectra without and with four increasing levels of added substrate. *Contour colors* are set by the two-letter-code *ct* (with 0 for *red*, 1/8 for *tomato*, 1/4 for *magenta*, 1/2 for *blue*, 1 for *green*). In the *Titration Plot* window, by choosing the spectra and the observable to be compared (in this case, the ^1^H chemical shift of H96) and clicking the *Plot* button, *NDPPlot* graphs ^1^H NMR chemical shift of H96 as a function of the molar ratio. **c** The *Peak Height Analysis* tool (two-letter-code *rh*) can be used to analyze results from relaxation experiments or any other peak intensity related experiments. After choosing a series of assigned spectra with different time/condition parameters, clicking the *Plot T*-*decay* button can be used to visualize the relaxation time constant as a function of residue number or by choosing a single residue, the decay in its peak intensity over time/condition. Alternatively, *per residue* and *per spectrum* intensity analysis options are available for observing overall differences between residues or spectra

**Fig. 7:**
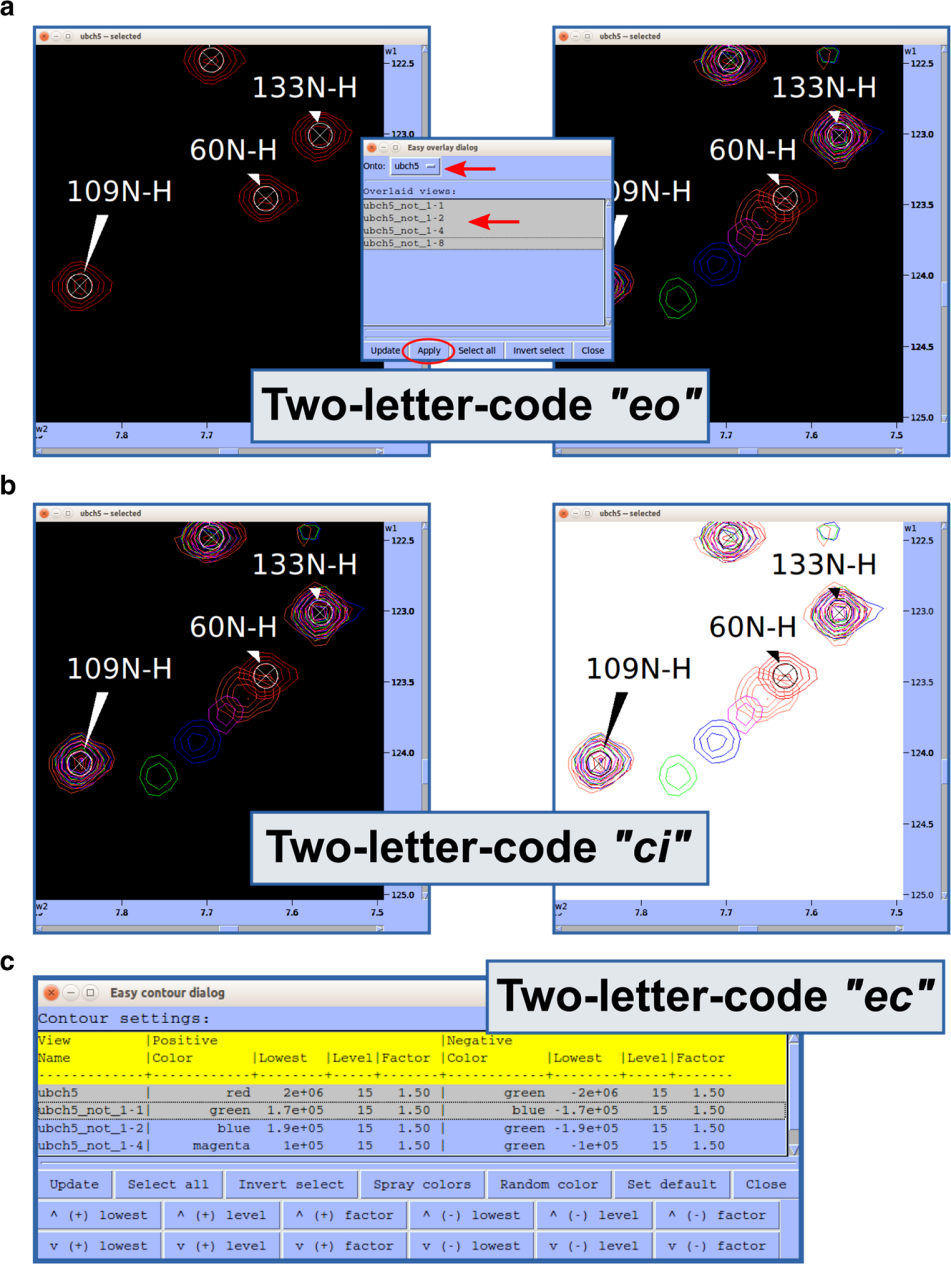
Useful tools for spectral series analysis. **a** Easy overlay dialog. We developed much simpler tool for spectral overlay than traditional “ol” tool particularly for many spectra. It is activated by two-letter-code “eo”. Simply, user can choose a spectrum “onto” and select spectra to “overlay” by mouse dragging and clicking with Ctrl and Shift keys. When most of all spectra in the project need to be overlaid, user can simply click Select all button and exclude spectra by Ctrl key and mouse clicks. **b** Change inverse background. We understood the visual limitation of black background color for spectral series analysis if user needs to color spectra differently. Thus, we developed two-letter-code “ci” to change background color from black to white and from white to black. The background color can be set differently and if ornament color is black and white, they are automatically changed to the other color, and so markers such as crosshair are. **c** Easy contour dialog. This tool is activated by two-letter-code “ec”. It can be used to adjust contour color, level and threshold of multiple spectra all at once

The graphical chart tool, *NMR Data Perturbation Plot (NDPPlot)*, which was originally an internal chart module of *Ponderosa Analyzer*, has been isolated from the program to be an independent program and also integrated into *NMRFAM*-*SPARKY*. *NDPPlot* supports seamless visualization of a series of NMR spectra, such as time series or titrations. *Perturbation Plot* (two-letter-code *np*, Fig. 6a) displays global spectral changes resulting from a change in solution conditions or composition. *Titration Plot* (two-letter-code *ni*, Fig. 6b) traces changes in the chemical shift of a particular resonance as the function of a variable such as pH or added ligand. *Peak Height Analysis* (two-letter-code *rh*, Fig. 6c) is used in the analysis of data for relaxation measurements. With a few clicks (*Save to graphics* button), *NDPPlot* is capable of generating figures and plots in the popular scalable vector graphics format (SVG). The *NDPPlot* program accepts INI (ititialization) format as input and saves graphics files. It includes useful mouse functions, such as entity identification, zoom in, zoom out and pan. This program is designed for visualizing and analyzing spectral series data; however, we started providing *NDPPlot* compatible files from our *PINE* and *PECAN* web servers because we found that the zooming capability of *NDPPlot* improved the visualization of data from larger proteins. Because the traditional overlay dialog (two-letter-code *ol*) is limited to overlaying one spectral view at a time, we added *Easy overlay dialog* (two-letter-code *eo*), which lets users select multiple spectral views for overlay onto a specified view (Fig. 7a). A white background, which is better for visualizing differently colored data from multiple spectra, can be selected (two-letter-code *ci*, Fig. 7b). The *Easy contour dialog* (two-letter-code *ec*, Fig. 7c) box enables the adjustment of contour threshold, levels, and colors for multiple spectra.

#### Secondary structure prediction

**Fig. 8:**
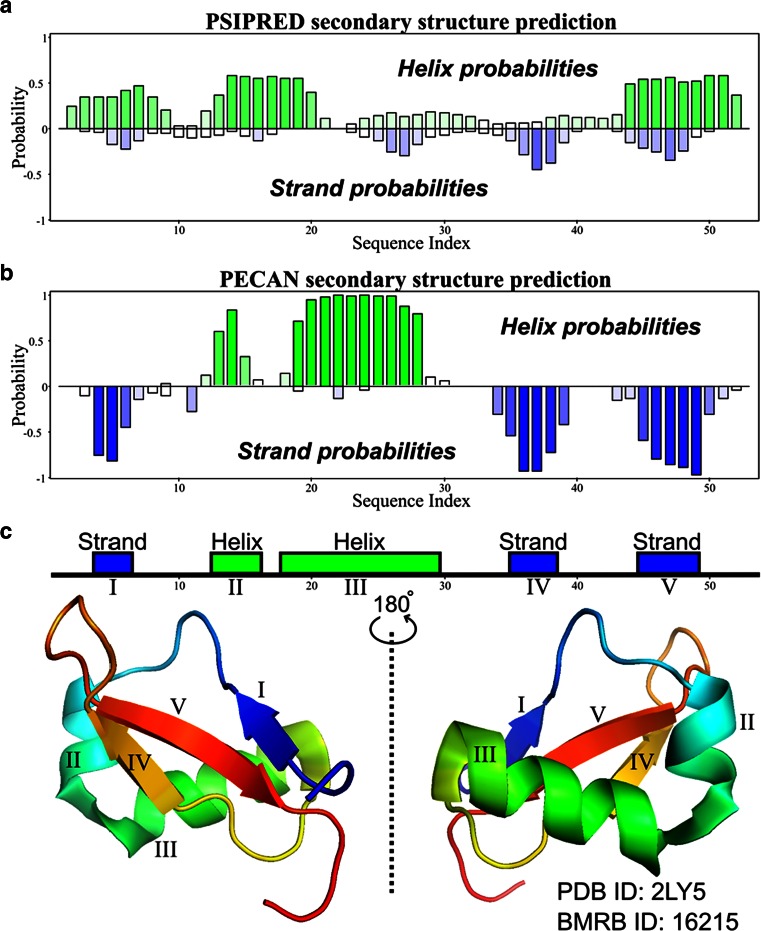
Sequence-based and chemical shift-based predictions from *NMRFAM*-*SPARKY* of the secondary structure of the protein brazzein (PDB ID: 2LY5, BMRB ID: 16215). The illustration was made by *NDPPlot*. **a** Sequence-based prediction by *PSIPRED* (two-letter-code *PP*) could not predict secondary structures correctly and the probabilities are not confident. **b** Chemical shift-based prediction by *PECAN* (two-letter-code *n6*). **c** Secondary structures from PDB deposited structure. The *PECAN* prediction matched perfectly with the secondary structural elements determined from the structure

*NMRFAM*-*SPARKY* supports both sequence-only (*PSIPRED)* (Jones et al. 1999) and chemical shift-based methods (*PECAN*) (Eghbalnia et al. 2005) or (*TALOS*-*N*) (Shen and Bax 2013) for secondary structure prediction. Generally, sequence-only methods yield 70–80 % accuracy, and the accuracy can be improved by using chemical-shift-based methods (Fig. 8). For example, we determined that *PECAN* surpassed *PSIPRED* in predicting the secondary structure of the small protein brazzein (PDB ID: 2LY5, BMRB ID: 16215) (Cornilescu et al. 2013). *Ponderosa Server* is a part of the *PONDEROSA*-*C/S* package (Lee et al. 2014) that automatically runs *TALOS*-*N* and applies optimized torsion angle constraints for the structure calculation. *Ponderosa Analyzer*, another component of *PONDEROSA*-*C/S*, offers tools for refining torsion angle constraints.

#### Three-dimensional structure prediction of proteins

**Fig. 9:**
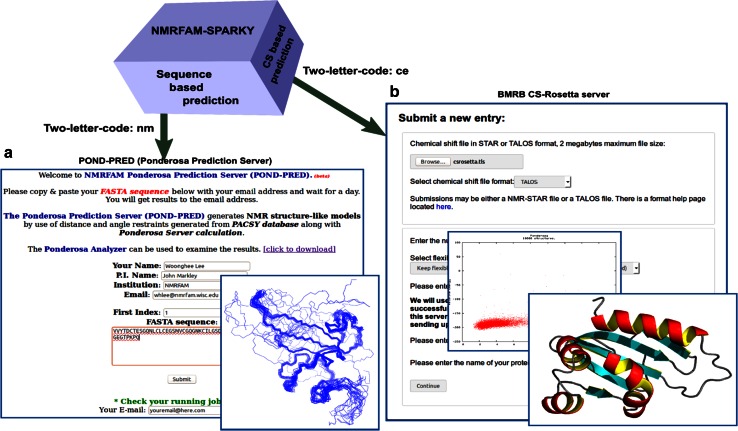
Three-dimensional structures can be predicted by sequence-based method and chemical shift-based methods in *NMRFAM*-*SPARKY*. **a** *POND*-*PRED* (two-letter-code *nm*) is a webserver offered by NMRFAM for predicting protein 3D structures from amino acid sequences. **b** *CS*-*Rosetta* is a chemical shift-based 3D structure prediction program; it is accessibly from *NMRFAM*-*SPARKY* (two-letter-code *ce*) on a web server hosted by BMRB

*Integrative NMR* supports predictions of protein 3D structure either on the basis of amino acid sequence alone and on the basis of assigned NMR chemical shifts. Jobs to be carried out on external servers are launched from NMRFAM-SPARKY. The sequence-only method, *POND*-*PRED* (Ponderosa Prediction Server), which is carried out on an NMRFAM server (http://ponderosa.nmrfam.wisc.edu/model.html), predicts hydrogen bond constraints from PSIPRED results and analyzes the *PACSY* database to generate distance and angle constraints. This method generates structures by simulated annealing as in typical NMR structure calculations (Fig. 9a). The chemical-shift-based method utilizes *CS*-*Rosetta* calculations (Shen et al. 2008) carried out on a server at BMRB (https://csrosetta.bmrb.wisc.edu/csrosetta) that employs the *Condor* (Thain et al. 2003) grid computing system (Fig. 9b).

### PONDEROSA-C/S

#### Three-dimensional structure determination

**Fig. 10:**
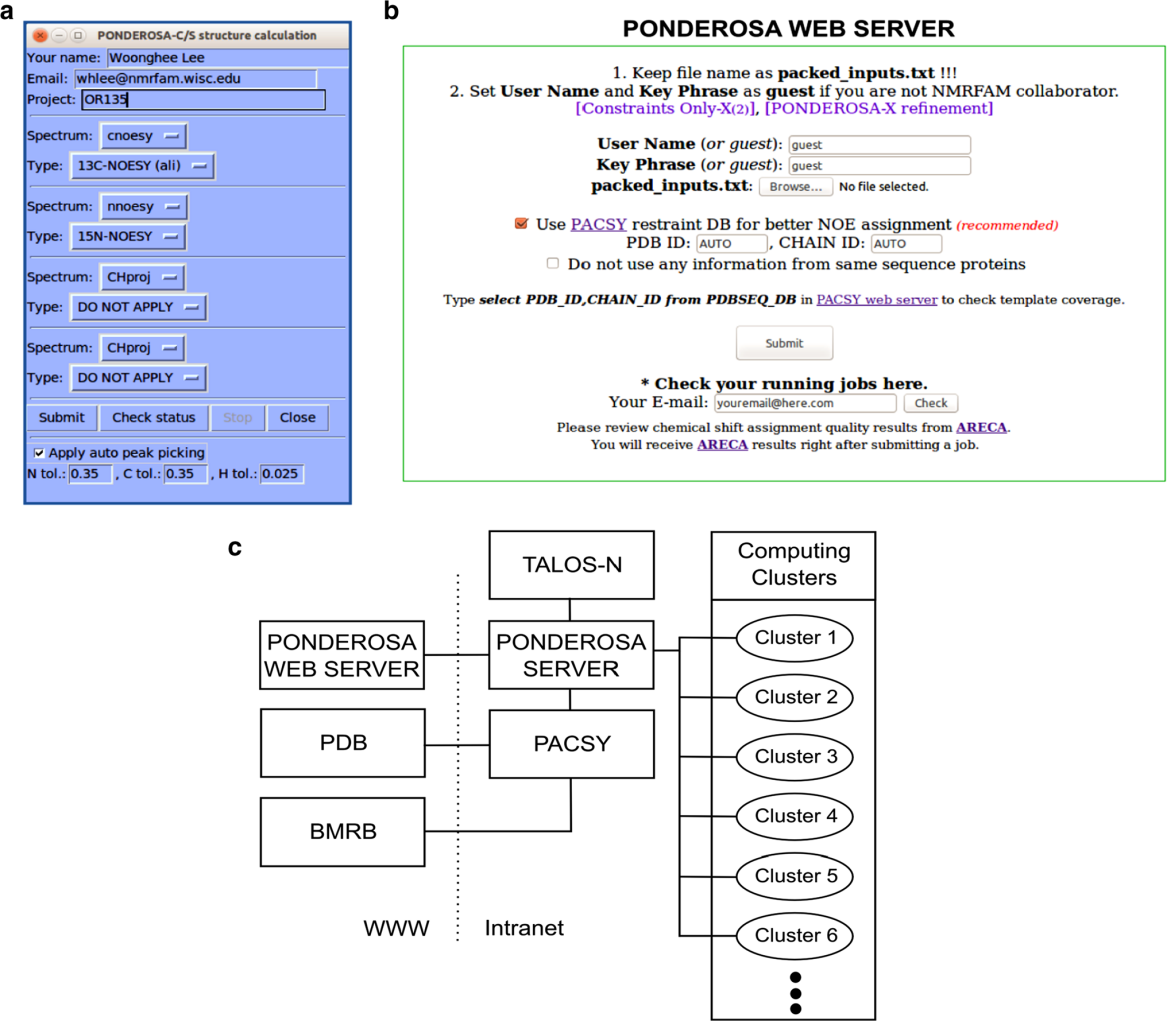
NMR-based 3D protein structure calculation. **a** Calculation 3D structure by *PONDEROSA*-*C/S* (two-letter-code *c3*) offers direct job submission from *NMRFAM*-*SPARKY* to the *Ponderosa Web Server*. It supports fully automated mode with and without automated NOESY peak picking and semi-automated mode with partially or fully assigned NOESY data. **b** *Ponderosa Web Server* is a freely available web resource that transmits the structure calculation command to the *Ponderosa Server* running at NMRFAM. **c** Diagram showing the integrated architecture of *Ponderosa Web Server* and *Ponderosa Server* at NMRFAM

Integrative NMR supports a complete environment for structure calculation. The initial version of *PONDEROSA* demonstrated its potential by generating accurate structures from raw NOESY spectra in the second round of the CASD-NMR competition (Rosato et al. 2015). The newer version, *PONDEROSA*-*C/S*, that is part of *Integrative NMR* isolates the computation module on a server allowing the user to focus on the input and output data. The integration of *NMRFAM*-*SPARKY* with *PONDEROSA*-*C/S* makes it possible to calculate and verify structures with a few clicks. For example, a new structure calculation (two-letter-code *c3*) requires only clicking to specify the assignment file and raw NOESY spectra and entering the user’s e-mail address (Fig. 10a). Then, after clicking the ‘Submit’ button, NOE cross peaks from the spectra are picked and evaluated, and a pre-packed *Ponderosa Server* input file is sent to the *Ponderosa Web Server* for structure determination (Fig. 10b). Details are provided below.

#### Ponderosa web server

The *Ponderosa web server* is a free computational resource for structure calculation (Fig. 10b, http://ponderosa.nmrfam.wisc.edu/ponderosaweb.html) maintained by NMRFAM. The server benefits from monthly updates of the *PACSY DB* and offers the most recent version of the *Ponderosa Server* software (Fig. 10c). As a default, the structure calculation utilizes *Xplor*-*NIH* and includes the *AUDANA* algorithm and water refinement. The final stage of the structure determination calculates 100 structures with constraints obtained from AUDANA by setting the option to *Constraints only for final step* in the Ponderosa Client program (Lee et al. submitted).

#### Ponderosa client

**Fig. 11:**
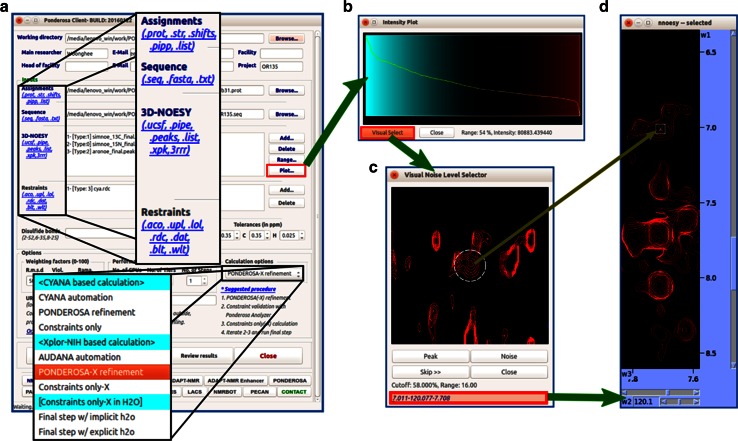
*Ponderosa Client* supports several formats and settings for structure calculation. **a** *Ponderosa Client* accepts several types of input including NOE (raw spectra: .ucsf, .pipe; peak list: .peaks, .list, .xpk, 3rrr), RDC (.rdc), and SAXS (.dat) for Xplor-NIH based structural calculations. **b** The optimized noise threshold for NOESY peak picking is determined automatically by *Intensity Plot*, which ranks the intensities of NOE peaks and uses an *r* ^−6^ (r: distance between two protons) approach to estimate the intensity corresponding to the 5.5 Å cutoff (*blue* robust range, *black* mixture of real peaks and noise, *red* noise range). **c** Alternatively, the user can employ the *Visual Select* tool to determine the noise level. This tool randomly selects a position where the chemical shift assignments suggest that a peak should be found, and the user decides whether the signal is a real peak or noise. **d** The *Visual Select* tool is integrated with *NMRFAM*-*SPARKY* for better decision making. Clicking the peak position in the Visual Select tool enables the user to navigate (two-letter-code *up*) to the position of the peak in a spectrum displayed by *NMRFAM*-*SPARKY*

The *Ponderosa client* accepts a wide range of inputs in addition to NOESY spectra. Also supported are: residual dipolar coupling (RDC), small angle X-ray scattering (SAXS), and wide angle X-ray scattering (WAXS) data (Fig. 11a). Manual constraints can be added and combined with automated NOE assignments. *Intensity Plot* automatically analyzes the intensities from long range peaks and uses an *r*^*−*6^ approximation to predict the 5.5 Å intensity threshold (Fig. 11b); signals beyond this threshold are considered to be noise.

The *Visual Select* tool in *Intensity Plot* (*NMRFAM*-*SPARKY* two-letter-code *up*; Fig. 11c) supports more refined manual noise threshold adjustment. It visualizes positions at which real peaks are predicted to appear at a certain threshold and allows user to decide whether the data support a real peak. The user is guided to find the optimal noise threshold level by a few clicks. This feature also can be used to determine positions of peaks in overlapped regions of strip plots (Fig. 11d).

#### Ponderosa server

Structure determination jobs are submitted to the *Ponderosa Server*. Once the calculations are completed, the user is sent an email containing the URL from which the results can be downloaded. We keep upgrading the program and installing in NMRFAM servers. Thus, a user using our server always uses the latest version at the time without any other installation.

#### Ponderosa analyzer

**Fig. 12:**
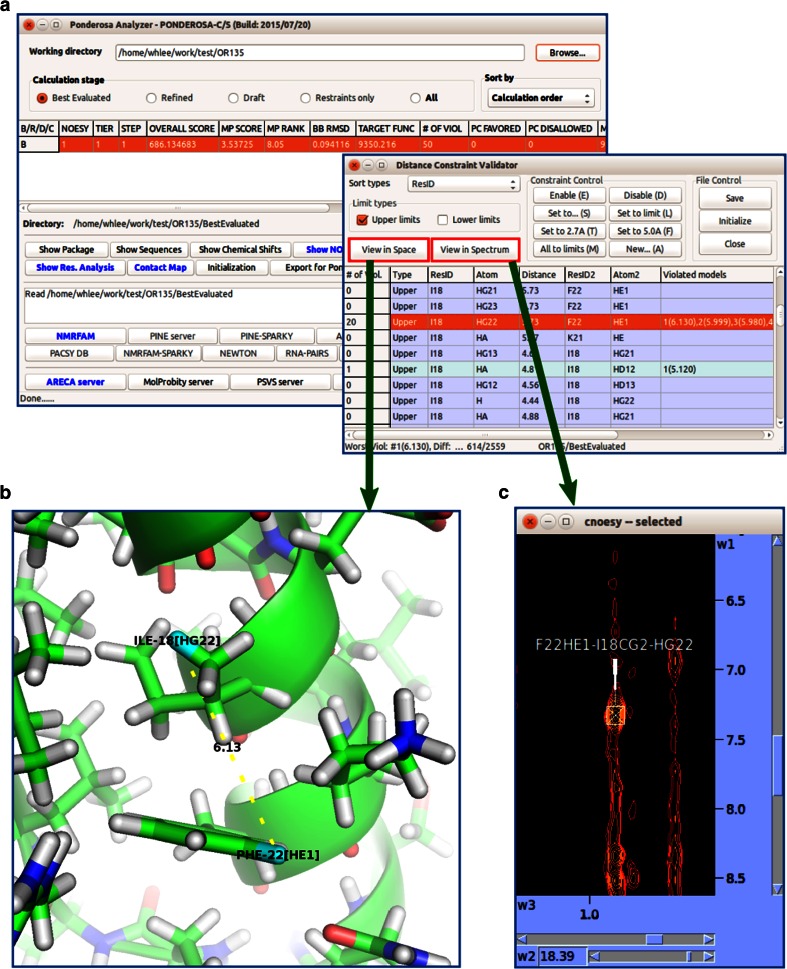
*Ponderosa Analyzer* provides tools for validating assignments, constraints and structures that are integrated with *Enhanced PyMOL* and *NMRFAM*-*SPARKY*. **a** *Distance Constraint Validator* is a tool for analyzing distance information extracted from NOESY data. **b** 3D illustration of the constraint selected (*Enhanced PyMOL* command *@p*). **c** NOESY spectrum highlighting the experimental evidence for the selected constraint (*NMRFAM*-*SPARKY* two-letter-code *up*). The user can manually adjust or exclude the examined constraint for the next run by means of the *Constraint Control* buttons in the *Distance Constraint Validator*

*Ponderosa Analyzer*, which integrates an enhanced version of *PyMOL* and *NMRFAM*-*SPARKY* (Fig. 12), is designed to analyze not only coordinates but also essential characteristics of the protein. *Enhanced PyMOL* is activated by launching regular *PyMOL* from *Ponderosa Analyzer*, which includes several tools described below that can be used to refine the input used for structure determinations.

#### Constraint validation

*Distance constraint validator* (Fig. 12a) is a validation tool for distance information extracted from NOESY data that integrates *Enhanced PyMOL* (command *@p*, Fig. 12b) and *NMRFAM*-*SPARKY* (two-letter-code *up*, Fig. 12c). *Distance Constraint Validato*r enables the user to exclude or adjust erroneously extracted inter-proton constraints by simply clicking buttons in the control panel of the program. *Ponderosa Violation Investigator(Ponderosa VI)* is a simplified validator that runs independently from *Ponderosa Analyzer* and supports quick violation lookup.

#### Hydrogen bond constraints

**Fig. 13:**
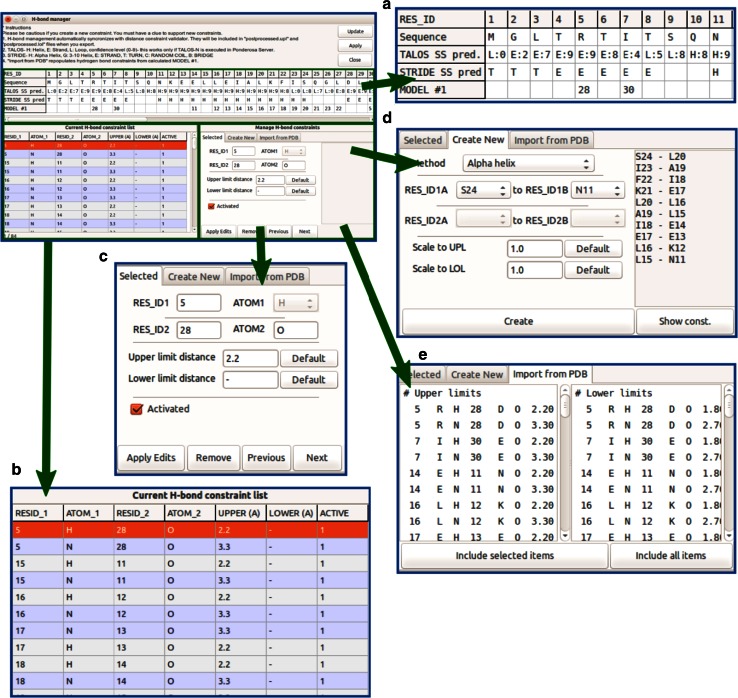
*H*-*bond manager* is a Ponderosa Analyzer tool that provides an easy way to add or remove hydrogen bond constraints. **a** The *H*-*bond manager* panel shows secondary structure information from *TALOS*-*N* prediction and from close distances detected structural models from a previous calculation. **b** Current hydrogen bond constraints are listed in the *lower-left panel*. During the first structure calculation, they are generated automatically from NOE cross peak patterns. The updates in the *lower-right panel* change the content in this panel. **c** An H-bond constraint selected in the *lower-right panel* can be modified or removed. **d** New constraints from characteristic secondary structures can be easily added. **e** Close atom distances from the most recent structure determination can be reviewed as possible H-bonds and can be added as constraints for the next structure determination

With *H*-*bond Manager* (Fig. 13), the user can add or remove hydrogen bond information on the basis of experimental H/D exchange data, characteristic NOE patterns, patterns of secondary chemical shifts, trans H-bond couplings, or results from previous calculations.

#### Management of constraint types

**Fig. 14:**
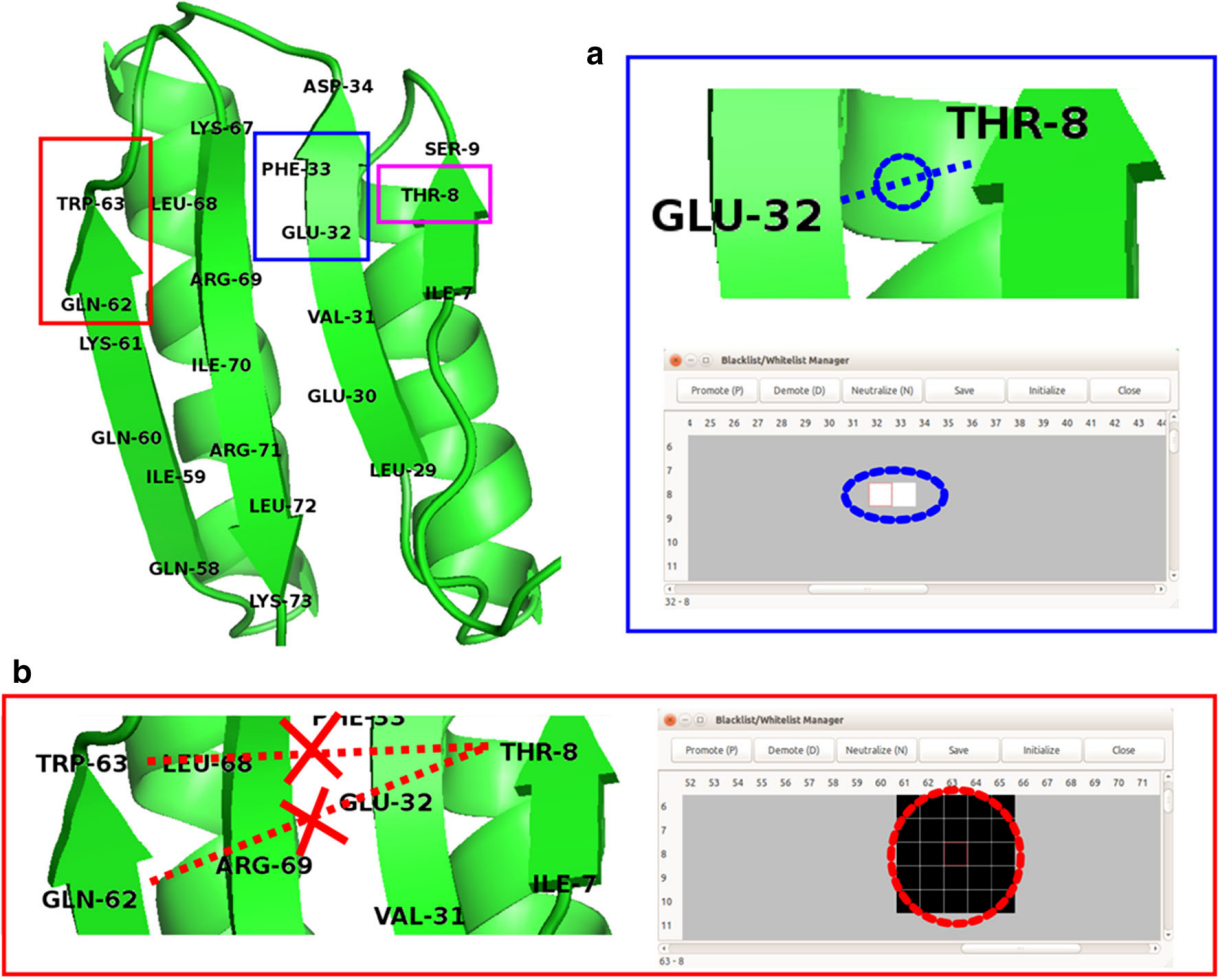
*Blacklist* and *Whitelist* are constraint types supported exclusively by *PONDEROSA*-*C/S* for resolving ambiguity in NOESY data by applying different weighting factors to the inter-residue contacts. The *Blacklist/Whitelist Manager* provides a graphical user interface to change the weighting of individual residue–residue contacts. **a** As an example, if the user is certain that T8-E32 and T8-E33 are close enough to have NOE cross peaks, the corresponding grids can be promoted and *colored white*. **b** If the user is certain that T8-W63, T8-Q62 and the surrounding residues are too distant to produce NOE cross peaks, the corresponding grids can be blacked out to avoid errors in automated NOE assignment and structure calculation by the *Ponderosa Server*

The *Blacklist/Whitelist Manager* (Fig. 14) provides a graphical user interface that enables the user to modify the weighting factors of inter-residue contacts. For example, if the user determines that two protons are close enough to have an NOE cross peak, the weighting can be *promoted*. Alternatively, if an NOE connectivity is determined to be erroneous, its weighting can be *demoted*. Revised constraint files are automatically generated when the user selects ‘*Export to the Ponderosa Client*’ in the main window of *Ponderosa Analyzer.*

#### Analysis of contacts

**Fig. 15:**
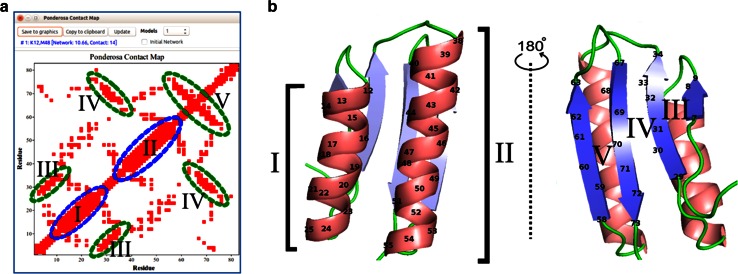
*Contact Map* is a *Ponderosa Analyzer* tool that assists with structural and spectral analysis of the protein. Because it displays inter-proton distances shorter than 5.5 Å, unlike maps that simply show C^α^–C^α^ contacts, it can be used to predict cross peaks in NOESY spectra. **a** *Contact Map* displays patterns that identify secondary structure. **b** Cartoon representation of the 3D structure of the protein with secondary structural elements colored (*Enhanced PyMOL* commands *@sc* and *@cs*). Good agreement is seen between the secondary structural elements shown in **a** and **b** (I and II, alpha helices; III, parallel sheet; IV, anti-parallel sheet consisting of two strands far apart in the sequence; V, anti-parallel sheet consisting of two strands close in the sequence connected by a short turn)

*Contact Map* illustrates residue–residue contacts from a three-dimensional structural model as a simple two-dimensional plot that reveals secondary structural features (Fig. 15). *Contact Map* can be used to identify inter proton distances that are shorter than 5.5 Å and are predicted to give rise to NOE cross peaks.

#### Analysis of backbone dihedral angles

**Fig. 16:**
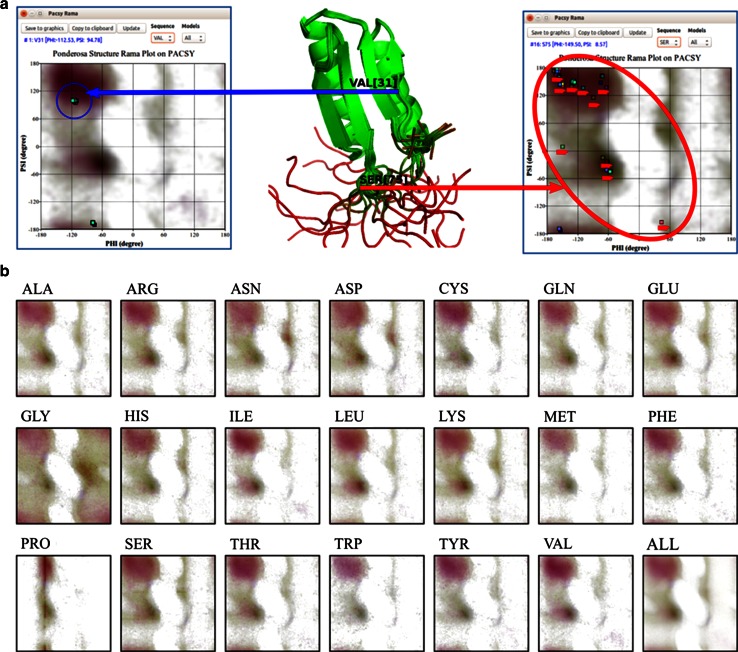
*Pacsy Rama* provides a set of images, derived from quantitative analysis of φ and ψ angles of proteins in the PACSY database, that provide a visual representation of favorable dihedral regions according to amino acid type or for all amino acid types. The *images* are useful for assessing the structural quality and dynamic characteristics of NMR solution structure of a protein. **a** In this example, the φ/ψ angles for V31 in the 20 models representing the structure, are closely clustered in an energetically favorable region, whereas the φ/ψ angles for S75 from the various models are highly dispersed consistent with the residue being present in an ill-defined region of the protein. **b** *Pacsy Rama* images for the 20 common amino acids and all combined. These images are downloadable from (http://pine.nmrfam.wisc.edu/download_packages.html)

Plotting backbone φ/ψ dihedral angles onto a Ramachandran plot provides a useful way of assessing structural quality. A ‘good’ structure is expected to have backbone dihedral angles clustered in the statistically favorable areas, and outliers may be indicative of errors or the presence of forces that perturb the structure to a higher energy state. In addition, large deviations provide evidence for structural flexibility, such as that for S24 in the example shown in Fig. 16a. *Pacsy Rama* is a tool that enables the user to display the φ/ψ values for residues in a set of structural models against a consensus Ramachandran plot or a Ramachandran plot for the specific residue type (Fig. 16b). Specialized Ramachandran plots were derived from the PACSY database by counting the occurrences of dihedral angles within 4° × 4° φ/ψ voxels restricted by secondary structure type or amino acid type. From these, image files were created for each of the 20 standard amino acid residues and also for the consensus. The images, which are in PNG (Portable Network Graphics) format, are downloadable from (http://pine.nmrfam.wisc.edu/download_packages.html).

#### Analysis of distance constraints

**Fig. 17:**
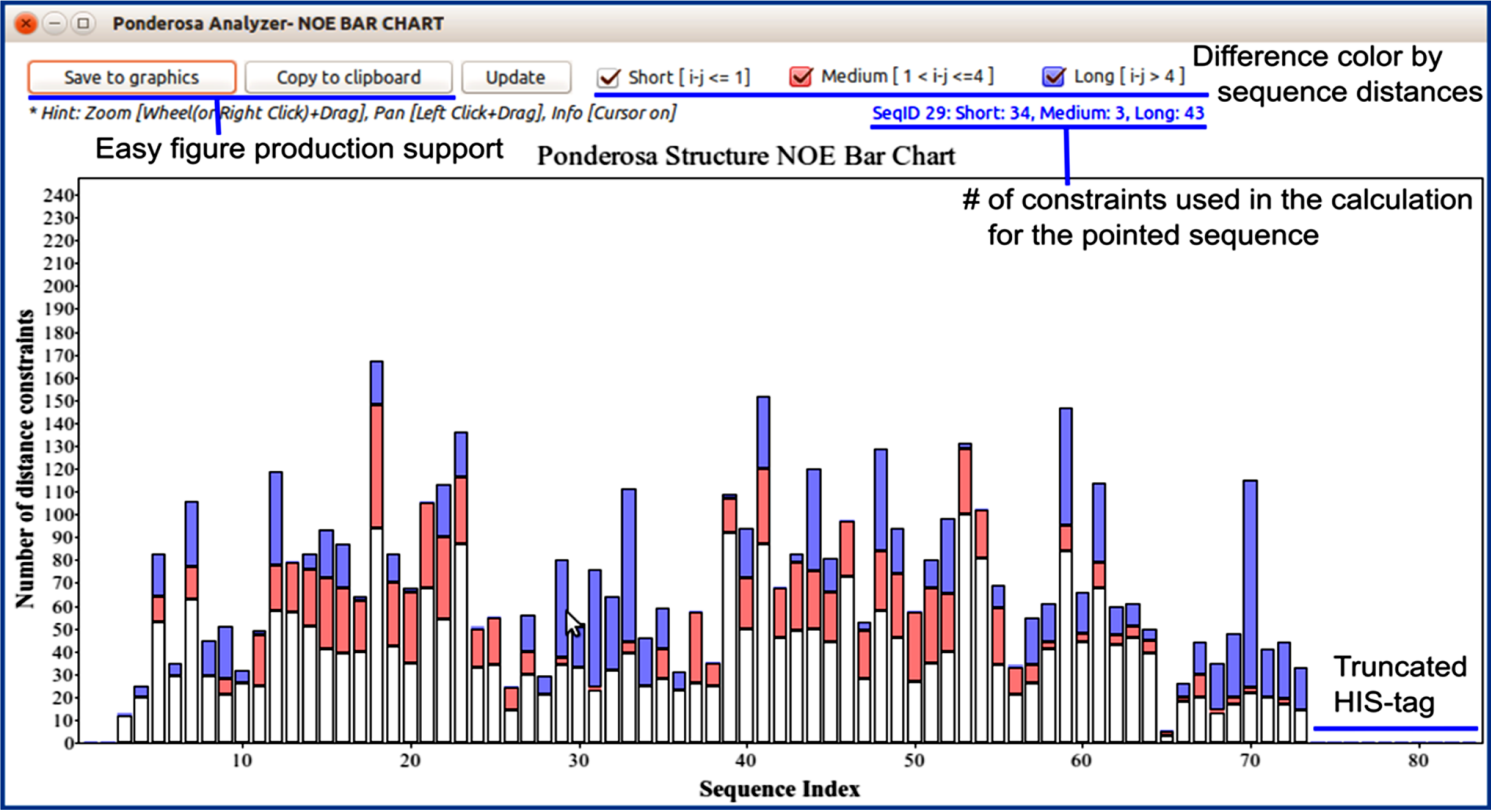
The *NOE Bar Chart* tool in *Pacsy* Analyzer represents the number and type (*white* short range; *red* medium range; *blue* long range) of distance constraints for each residue used in a structure calculation

**Fig. 18:**
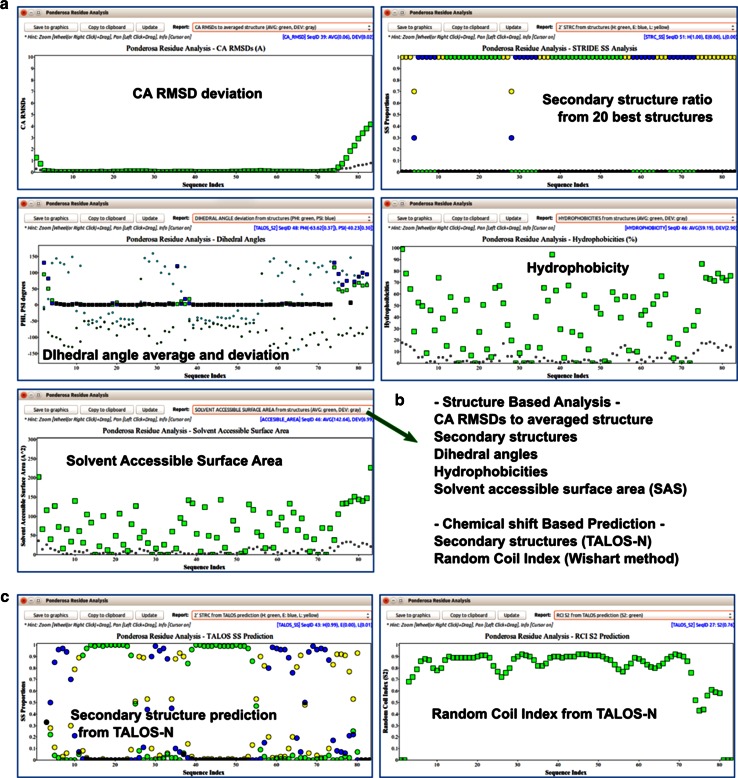
The *Residue Analysis* tool in *Ponderosa Analyzer* provides visual representation of the properties of amino acid residues plotted by sequence number. **a** Examples of structure-based properties derived from the 20 best models from the structure determination. **b** List of the features supported by structure-based analysis and chemical shift-based prediction. **c** Examples of chemical shift-based predictions. Secondary structure and random coil index (S2) values from *TALOS*-*N* based on backbone chemical shifts. The user can compare properties predicted from the backbone chemical shifts with those from the calculated structures

**Fig. 19:**
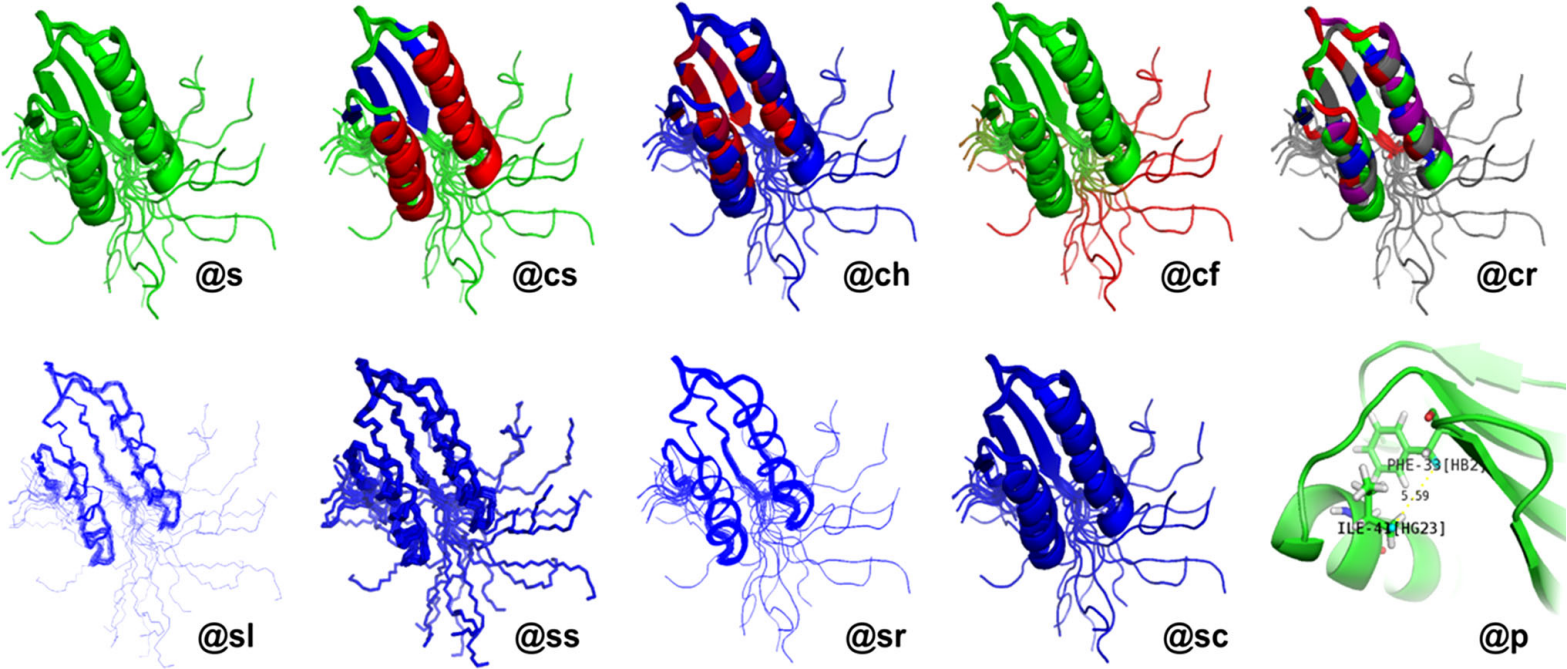
*Ponderosa Analyzer* offers these 10 preset drawing modes in *Enhanced PyMOL* as assigned by the shortcut codes shown. See Table 2 for descriptions

*NOE Bar Chart* is a tool that shows the number of distance constraints for each residue used in the structure calculation (Fig. 17). These numbers provide an indication of the quality of the structure and identify regions that are ill-defined. The results may indicate that more effort needs to be expended in identifying additional distance constraints. The *Cα RMSD Chart* and *Random Coil Index Prediction Chart* (Fig. 18a) can be used to identify disordered regions. In addition, the *Color by Flexibility* command (*@cf*) for *Enhanced PyMol* can be used to distinguish well-defined from ill-defined regions of the protein (Fig. 19; Table 2). Additional information about internal mobility may be available from cross-relaxation and relaxation results. If heteronuclear NOE data are available, they can be visualized in *NMRFAM*-*SPARKY* by means of *Perturbation Plot* (two-letter-code *np*, Fig. 7a), and if *T*_1_/*T*_2_ relaxation data have been collected, they can be visualized using *Peak Height Analysis* (two-letter-code *rh*, Fig. 6c).

**Table 2:** Shortcut commands used by *Enhanced PyMOL* for custom visualization

Command	Description
@s	Split models in the ensemble
@cs	Color by secondary structure (red: helix, blue: strand, green: loop)
@ch	Color by hydrophobicity (red: <10 %, blue: ≥30 %, gradient from red to blue between 10 and 30 %)
@cf	Color by flexibility (red: ≥2.0 Å for average Cα RMSD, gradient from green to red between 0 and 1.5 Å)
@cr	Color by magnitude of RDC violation (red: ≥4.5 Hz, purple: 3.0–4.5 Hz, blue: 1.5–3.0 Hz, green: <1.5 Hz)
@sl	Show backbone as lines
@ss	Show backbone as sticks
@sr	Show backbone as ribbon
@sc	Show backbone as cartoon
@p	Display selected distance/angle constraints from Ponderosa Analyzer

#### Secondary structure analysis

The *Residue Analysis* tool is a visual chart tool for easy recognition of structural properties on a residue basis. *Residue Analysis* supports structure-based analysis (Fig. 18a) and chemical-shift-based prediction (Fig. 18c). For the best 20 models from the structure calculation, the structure-based analysis provides a visualization of C^α^ atom RMSDs to the average structure, secondary structure, dihedral angles (φ and ψ), hydrophobicities, and solvent accessible surface area (SAS). Chemical-shift-based prediction provides a visualization of secondary structure and random coil index derived order parameters (*S*^2^) predicted from *TALOS*-*N* (Fig. 18b). Comparison of the results can yield insights about the quality of the structure determination and the particular characteristics of the protein.

#### RDC analysis

**Fig. 20:**
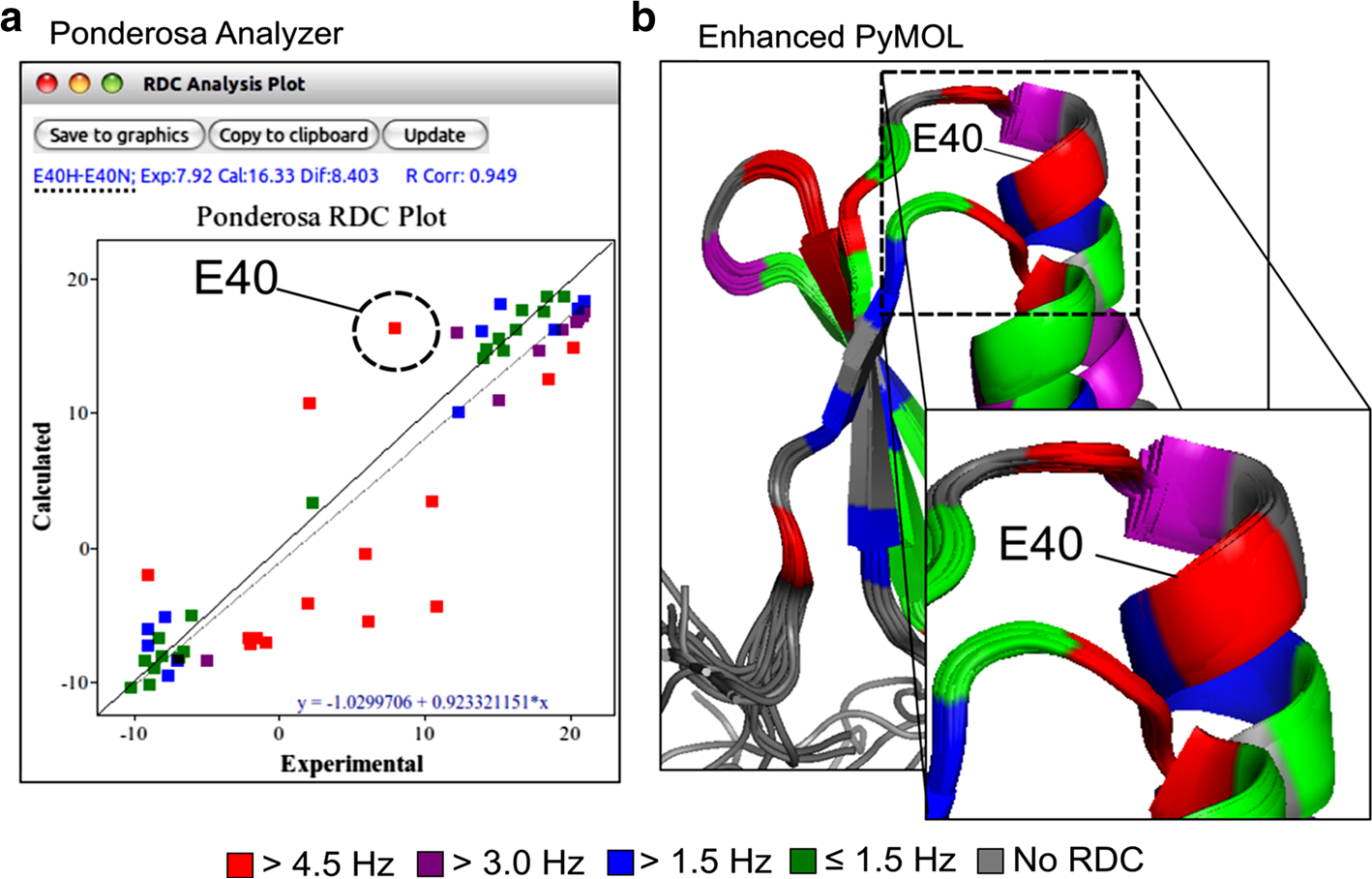
*RDC Analysis Plot* tool in *Ponderosa Analyzer*. **a** The *RDC Analysis* tool plots experimental RDC data versus RDC data calculated from the structure generated by *Ponderosa Server*. The linear least squares fitted line (*gray dashed line*) indicates the agreement between the experimental and calculated RDCs. **b** The shortcut command *@cr* (color by RDC violations) in *Enhanced PyMOL* uses the RMSD report generated by *Ponderosa Server* to visualize the correlation between experimental and calculated RDCs as a means for identifying potential errors in the 3D structure (or the in the RDC data)

RDC data provide global information about the orientations of individual bonds or entire secondary structure elements and can be used to validate or refine structures determined from NOESY data. This is particularly useful for all α-helical proteins or large proteins. RDC data can be included in the input to the *Ponderosa Server* and used to enhance automated NOE assignments. The *RDC Analysis* tool from Ponderosa Analyzer can be used to create a plot of experimental RDC data versus RDCs calculated from the structure (Fig. 20a). The linear least squares fitted line (gray dashed line) indicates the agreement between the experimental RDCs and the RDCs calculated from the structure generated by *Ponderosa Server*. *Enhanced PyMOL* (command *@cr*, Fig. 19) can be used to visualize the correlation between experimental and calculated RDCs and to depict potential errors in the 3D structure. In the illustration shown, residue E40 (colored in red) in the calculated structure does not agree with the input RDC data; thus, E40 is flagged both by the *RDC Analysis* tool in *Ponderosa Analyzer* (Fig. 20a) and by *Enhanced PyMOL* (Fig. 20b).

### Data visualization with enhanced *PyMOL*

As part of *Integrative NMR*, *Enhanced PyMOL* offers a range of data visualization options activated two- or three-letter shortcuts typed into the *PyMOL* command-line (Table 2; Fig. 19).

### Data output

Chemical shift assignments and peak lists generated by *Integrative NMR* can be outputted in NMR-STAR format (Markley et al. 2003) for direct deposition to the BMRB or wwPDB.

### Video tutorials

Structural analysis method described above for biomolecules are produced as video clips for any non-NMR expert to easily use NMR data in their research. They are freely available from the *NMRFAM*-*SPARKY* web page: (http://www.nmrfam.wisc.edu/nmrfam-sparky-distribution.htm), from the *PONDEROSA*-*C/S* web page: (http://ponderosa.nmrfam.wisc.edu/videos.html), or from the combined video playlist page: (http://pine.nmrfam.wisc.edu/integrative.html).

### Software availability and installation

All NMRFAM software is freely available from the NMRFAM Software Download page (http://pine.nmrfam.wisc.edu/download_packages.html). The *Integrative NMR* method requires the installation of *NMRFAM*-*SPARKY*, *Ponderosa Analyzer*, *Ponderosa Client* and *PyMOL*. The website provides instructions, installation scripts and video tutorials for their installation. The *NMRFAM Virtual Machine* is recommended for non-specialists, because it contains pre-installed versions of scientific software packages developed by NMRFAM and elsewhere, including *NMRFAM*-*SPARKY*, *Ponderosa Client*, *Ponderosa Analyzer*, *Ponderosa VI*, and *PyMOL* along with its Adaptive Poisson-Boltzmann Solver (APBS) plugin (Baker et al. 2001). A set of examples based on target OR135 from the second round of CASD-NMR is also included. The virtual machine (VM) can be run under a number of different virtualization software programs (VirtualBox and VMware among others) that support the Open Virtualization Format (.ovf, .ova). These virtualization programs are available for a wide variety of different popular host computers and operating systems (Windows, Mac OSX, Linux). A VM emulates a complete computer system. For example, the base operating system of the *Integrative NMR* VM is Ubuntu Mate 15.04 (64 bit Linux) (https://ubuntu-mate.org); the virtualization software allows this Linux VM to run natively on any host computer.
